# Effects of the Noncoding Subgenomic RNA of Red Clover Necrotic Mosaic Virus in Virus Infection

**DOI:** 10.1128/jvi.01815-21

**Published:** 2022-02-09

**Authors:** Pulkit Kanodia, W. Allen Miller

**Affiliations:** a Plant Pathology and Microbiology Department, Iowa State University, Ames, Iowa, USA; b Interdepartmental Genetics and Genomics Major, Iowa State University, Ames, Iowa, USA; c Plant Science Institute, Iowa State University, Ames, Iowa, USA; University of Maryland, College Park

**Keywords:** xrRNA, SR1f, XRN4, RCNMV, RNA-seq, virus-host interactions, *Tombusviridae*, xrRNA

## Abstract

In recent years, a new class of viral noncoding subgenomic RNA (ncsgRNA) has been identified. This RNA is generated as a stable degradation product via an exoribonuclease-resistant RNA (xrRNA) structure, which blocks the progression of 5′→3′ exoribonuclease on viral RNAs in infected cells. Here, we assess the effects of the ncsgRNA of red clover necrotic mosaic virus (RCNMV), called SR1f, in infected plants. We demonstrate the following: (i) the absence of SR1f reduces symptoms and decreases viral RNA accumulation in Nicotiana benthamiana and Arabidopsis thaliana plants; (ii) SR1f has an essential function other than suppression of RNA silencing; and (iii) the cytoplasmic exoribonuclease involved in mRNA turnover, XRN4, is not required for SR1f production or virus infection. A comparative transcriptomic analysis in N. benthamiana infected with wild-type RCNMV or an SR1f-deficient mutant RCNMV revealed that wild-type RCNMV infection, which produces SR1f and much higher levels of virus, has a greater and more significant impact on cellular gene expression than the SR1f-deficient mutant. Upregulated pathways include plant hormone signaling, plant-pathogen interaction, MAPK signaling, and several metabolic pathways, while photosynthesis-related genes were downregulated. We compare this to host genes known to participate in infection by other tombusvirids. Viral reads revealed a 10- to 100-fold ratio of positive to negative strand, and the abundance of reads of both strands mapping to the 3′ region of RCNMV RNA1 support the premature transcription termination mechanism of synthesis for the coding sgRNA. These results provide a framework for future studies of the interactions and functions of noncoding RNAs of plant viruses.

**IMPORTANCE** Knowledge of how RNA viruses manipulate host and viral gene expression is crucial to our understanding of infection and disease. Unlike viral protein-host interactions, little is known about the control of gene expression by viral RNA. Here, we begin to address this question by investigating the noncoding subgenomic RNA (ncsgRNA) of red clover necrotic mosaic virus (RCNMV), called SR1f. Similar exoribonuclease-resistant RNAs of flaviviruses are well studied, but the roles of plant viral ncsgRNAs, and how they arise, are poorly understood. Surprisingly, we find the likely exonuclease candidate, XRN4, is not required to generate SR1f, and we assess the effects of SR1f on virus accumulation and symptom development. Finally, we compare the effects of infection by wild-type RCNMV versus an SR1f-deficient mutant on host gene expression in Nicotiana benthamiana, which reveals that ncsgRNAs such as SR1f are key players in virus-host interactions to facilitate productive infection.

## INTRODUCTION

Long noncoding RNAs (lncRNAs) play important roles in diverse cellular processes, regulating gene expression during development, maintaining homeostasis, and responding to various abiotic and biotic stresses (1–9). Similar to the host counterparts during infection, some viruses make lncRNAs that are instrumental in regulating virus and host gene expression to modulate the virus life cycle and the host’s antiviral response (10–12). For instance, (i) polyadenylated nuclear (PAN) RNA is an lncRNA encoded by Kaposi’s sarcoma-associated herpesvirus (KSHV) that regulates viral gene expression (13), (ii) citrus tristeza virus (CTV) produces LMT1, an lncRNA that counteracts a plant’s defense response via inhibition of the antiviral salicylic acid signaling (14).

In contrast to the lncRNAs that are made during replication by the viral polymerase, a new class of viral lncRNA was identified over a decade ago that is generated by incomplete degradation of viral RNAs by the host’s cytoplasmic 5′→3′ exoribonucleases (XRNs) (15–19). The 5′→3′ XRNs in the nucleus and cytoplasm of eukaryotic cells are involved in RNA processing, RNA degradation, antiviral defenses, and regulation of gene expression, among other functions (20, 21). XRN1 is primarily present in the cytoplasm, while XRN2 is primarily present in the nuclei of yeast and metazoans (20, 21). The three known XRNs in plants, such as *Arabidopsis* AtXRN2, AtXRN3, and AtXRN4, are orthologs of XRN2 with only AtXRN4 being localized to the cytoplasm (22). Because no plant XRN with sequence homology to XRN1 has yet been identified, it is generally considered that AtXRN4 is a functional equivalent of XRN1 (20–22). The cytoplasmic 5′→3′ XRNs can use uncapped/decapped viral RNA as a substrate and function as an antiviral factor by degrading the viral RNAs from the 5′ end. However, all the viruses in the *Flavivirus* genus (15, 17, 23–26), several viruses in the *Luteoviridae*, *Tombusviridae* (16, 27–30), and *Benyviridae* (18, 19) families have evolved an XRN-resistant RNA (xrRNA) secondary structure in their viral RNA that can block the progression of host’s 5′→3′ XRN, resulting in xrRNA-derived subgenomic RNAs (sgRNAs). Putative xrRNA structures have also been identified *in vitro* in viruses belonging to the *Bunya-*, *Arena*-, *Betaflexi*-, *Virga*-, *Poty*-, and *Secoviridae* families (31, 32). Even though some xrRNA structures in the viral RNA have been identified in the intergenic region that can yield coding sgRNAs (29, 30, 33), most of the xrRNAs identified have been located in the 3′ untranslated region (UTR) that yield noncoding sgRNAs (ncsgRNAs) (15, 16, 18, 19, 24, 27–29, 31, 32, 34). The xrRNA-derived ncsgRNAs have been shown to play roles in virus pathogenicity, symptom development, virus movement and transmission, and suppressing the host’s antiviral responses (35, 36). For example, sfRNA from West Nile virus (WNV) and dengue virus (DENV) suppresses siRNA- and miRNA-induced RNAi pathways in insect and mammalian cells (37, 38), and DENV and Zika virus (ZIKV) sfRNA inhibits translation of interferon-stimulated genes (39–41), alters the stability of host mRNAs, and improves viral epidemiological fitness (42). Although flaviviral ncsgRNAs have been studied extensively, research on xrRNA-derived ncsgRNAs of plant viruses remains scarce. There is evidence that the xrRNA-derived ncsgRNA of beet necrotic yellow vein virus (BNYVV), called ncRNA3, partially complements the RNA silencing suppressor activity of BNYVV p14 protein and also facilitates systemic movement in plants (18, 19, 43).

**FIG 1:**
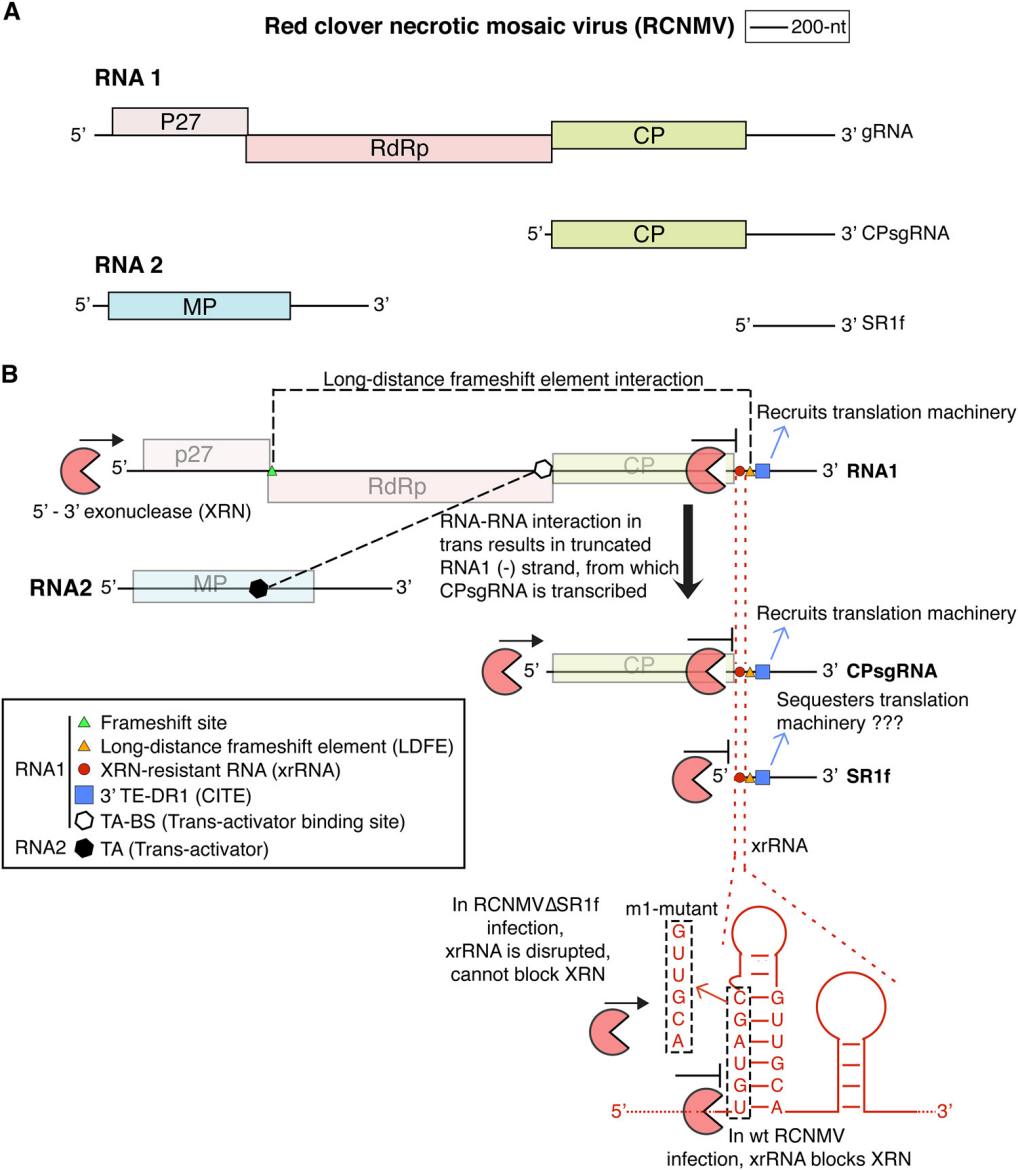
Genome organization of Red clover necrotic mosaic virus (RCNMV). (A) RCNMV genome map (drawn to scale) depicting the genomic RNAs (RNA1 and RNA2) and subgenomic RNAs (CPsgRNA and SR1f) produced during the infection. (B) Schematic diagram of some important RNA elements in RCNMV RNAs that are involved in cap-independent translation of RNA1, −1 programmed ribosome frameshift for the translation of p88 protein that contains the RNA-dependent RNA polymerase (RdRp) motif, synthesis of CPsgRNA by premature transcription termination, and production of SR1f via incomplete degradation of RNA1 and CPsgRNA by the host 5′→3′ exonuclease.

The first xrRNA-derived ncsgRNA that was discovered in a plant virus is SR1f of red clover necrotic mosaic virus (RCNMV) (16). RCNMV (genus *Dianthovirus*, family *Tombusviridae*) is a bipartite positive-strand RNA virus with genomic RNAs 1 and 2 (Fig. 1A) (44, 45). A subgenomic RNA (CPsgRNA1) that encodes the coat protein (CP) (Fig. 1A) is made from RNA1 via premature transcription termination during the negative-strand synthesis followed by positive-strand synthesis from the prematurely terminated transcription product (46). The xrRNA-derived ncsgRNA, SR1f (Fig. 1A), is generated as a stable degradation product formed by incomplete degradation of RNA1 and CPsgRNA1 by a still-unidentified 5′→3′ XRN (16, 27) (Fig. 1B). The 3′ UTR from which SR1f is derived controls both cap-independent translation, via its Barley yellow dwarf virus-like translation element (BTE) called TE-DR1 in RCNMV (47), and ribosomal frameshifting, via its long-distance frameshift element (LDFE) (48) (Fig. 1B). SR1f has been shown to *trans* inhibit both cap-independent and cap-dependent translation in cell-free translation extracts and in BY-2 protoplasts, possibly because the BTE (TE-DR1) binds key translation factor, eIF4F, thus making fewer copies of eIF4F accessible to host or viral mRNAs. This mechanism may explain the observation that SR1f indirectly *trans* inhibits negative-strand synthesis of RNA1 *in vitro* by repressing the production of the replicase protein (16).

Although SR1f is not absolutely required for a successful infection, cell-to-cell movement, and systemic movement of viral RNAs in Nicotiana benthamiana, the accumulation of RNA1 is significantly lower in plants infected with RCNMVΔSR1f mutant that is unable to generate SR1f (16). In addition, the functions of SR1f during RCNMV infection and how SR1f affects the plant on a molecular level are unknown. Understanding how SR1f affects host gene expression is important to determine its function, dissect the molecular mechanism by which it functions and discover a potentially novel strategy by which viruses can counteract plant defenses and stay a step ahead in the evolutionary arms race. In this study, we (i) assess RCNMV replication and symptom development in N. benthamiana and Arabidopsis thaliana, (ii) assess its role in counteracting the immune system, (iii) assess the role of host exoribonuclease XRN4 in generating SR1f, and (iv) perform a comparative transcriptomic analysis of N. benthamiana infected by wild-type RCNMV, which generates SR1f, and RCNMVΔSR1f, which does not make SR1f.

## RESULTS

### Symptoms and viral RNA accumulation in *N. benthamiana*.

**FIG 2:**
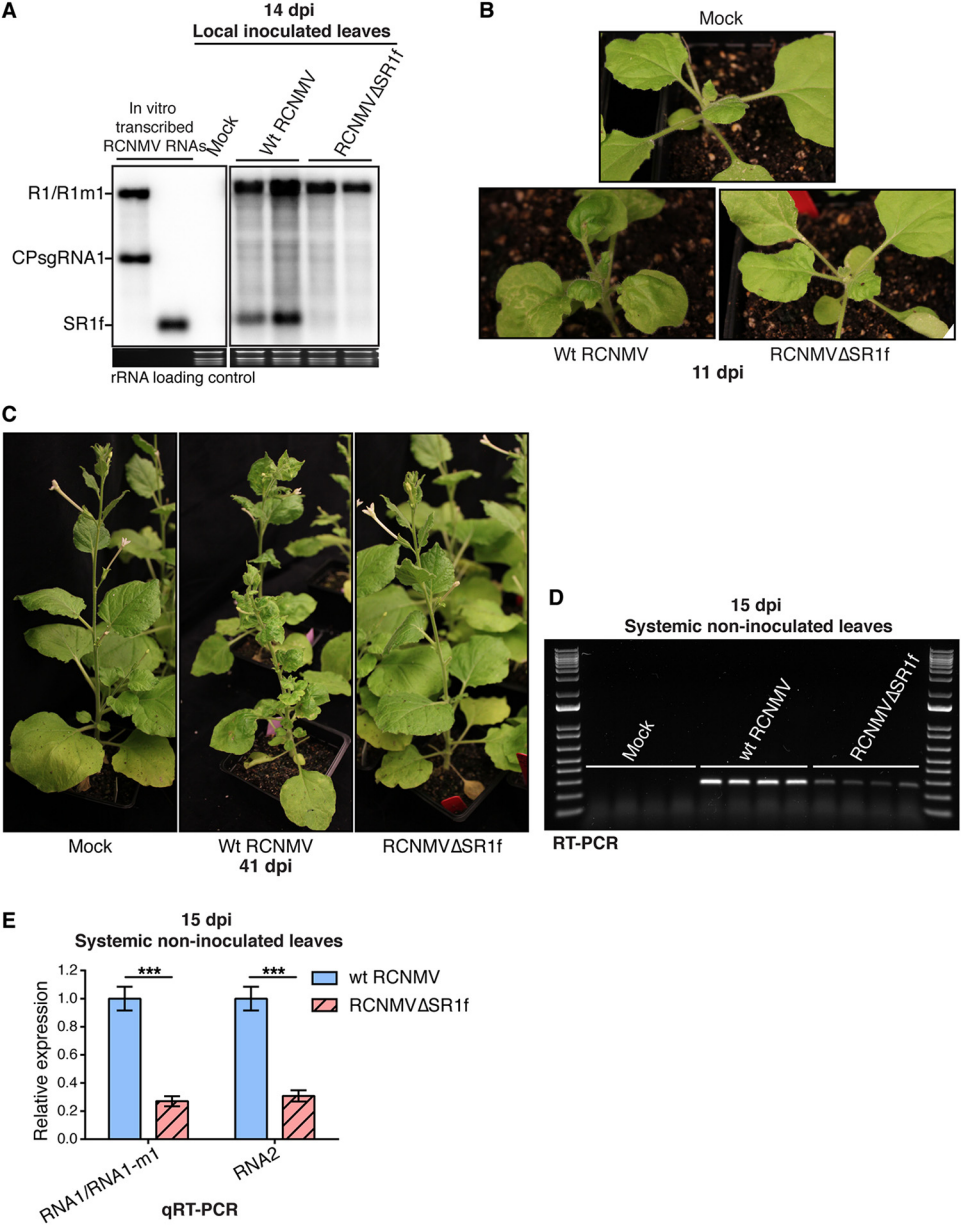
Effect of SR1f knockout on RCNMV infection in N. benthamiana. wt-RCNMV-infected plants were inoculated with wt RNA1 plus RNA2. RCNMVΔSR1f-infected plants were inoculated with RNA1-m1 plus RNA2. (A) Northern blot hybridization demonstrating that SR1f is produced only in wt-RCNMV-infected plants and not in RCNMVΔSR1f-infected plants, even though viral RNA accumulation was detected in both wt-RCNMV- and RCNMVΔSR1f-infected plants. SYBR Safe stained rRNA was used as loading control for the Northern blot. (B) Symptoms at 11 dpi. (C) Symptoms at 41 dpi. (D) RT-PCR to verify RNA1/RNA1-m1 accumulation. (E) qRT-PCR reveals relative amounts of each viral genomic RNA accumulated in wt-RCNMV- and RCNMVΔSR1f-infected plants. NbPP2A and NbL23 were used as reference genes for normalization of qRT-PCR data. ***, *P* < 0.001. Error bars indicate the standard errors of the mean (SEM).

To determine whether the presence of SR1f plays a role in symptom development, we inoculated N. benthamiana plants with (i) RCNMV RNA1 plus RNA2 (referred to as wild-type [wt] RCNMV), which generates SR1f, or (ii) RCNMV RNA1-m1 (Fig. 1B) plus RNA2 (referred to as RCNMVΔSR1f), which does not generate SR1f in local leaves (Fig. 2A) or systemic leaves (16, 49), as verified by Northern blot hybridization. RCNMV RNA1-m1 contains a six-base substitution (Fig. 1B) that disrupts the xrRNA structure in RNA1 at the 5′ end of its 3′ untranslated region (UTR) (16), and precludes SR1f production (49). At 11 days post-inoculation (dpi), necrosis, leaf curling, and mosaic symptoms were observed in wt-RCNMV-inoculated plants, while only very modest to no symptoms were observed in RCNMVΔSR1f-inoculated plants (Fig. 2B). Similarly, we observed severe symptoms in wt-RCNMV-inoculated plants but very modest to no symptoms in RCNMVΔSR1f-inoculated plants, even at later time points (Fig. 2C). In multiple independent experiments, same symptoms were observed even though both RCNMV RNA1 and RNA1-m1 accumulated in noninoculated systemic leaves in both inoculations, as verified by RT-PCR (Fig. 2D). Corresponding to the symptom phenotype and consistent with a previous report (16), viral RNAs accumulated to much lower levels in RCNMVΔSR1f infection compared to wt RCNMV infection, as verified by qRT-PCR (Fig. 2E). This indicates that the presence of SR1f contributes to symptom development and accumulation of viral RNAs during infection. Thus, the lack of symptoms in RCNMVΔSR1f-infected plants may be due to the reduced virus accumulation, and only a downstream, indirect effect of the lack of SR1f.

### Symptoms and viral RNA accumulation in *A. thaliana*.

**FIG 3:**
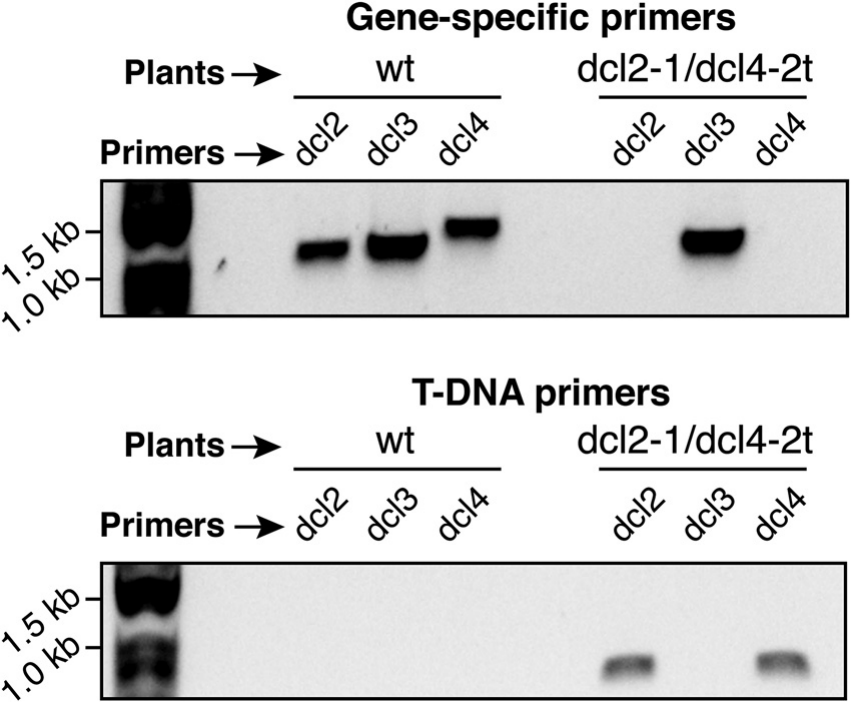
Genotyping *dcl2-1/dcl4-2t* double knockout mutant *Arabidopsis*. Gene specific primers refer to the forward and reverse primers for the respective *DCL* genes. T-DNA primers refer to the *DCL2* or *DCL3* reverse primer paired with the pROK2 vector forward primer, and the *DCL4* reverse primer paired with the pAC161_8474 vector forward primer. The primer sequences are listed in Table S4 in File 1 in the supplemental material.

We next tested how the presence of SR1f affects viral RNA accumulation and symptom development in wt Arabidopsis thaliana (Col-0), a model host plant with well-defined knockout mutations. Because the analogous flaviviral sfRNA1 in insect and mammalian cells and BNYVV ncRNA3 in plants have been shown to function as an RNA silencing suppressor (37, 38, 43), we hypothesized that RCNMV SR1f may also function as an RNA silencing suppressor. To test this hypothesis, we used *Arabidopsis dcl2-1/dcl4-2t* mutant. It is a double-knockout line with T-DNA insertions in *DCL2* and *DCL4* genes (50), which were verified as homozygous knockouts by PCR (Fig. 3). Among the four DCL proteins (DCL1, -2, -3, and -4) in *Arabidopsis*, DCL2 and DCL4 generate virus-derived siRNAs required for antiviral RNA silencing (51).

**FIG 4:**
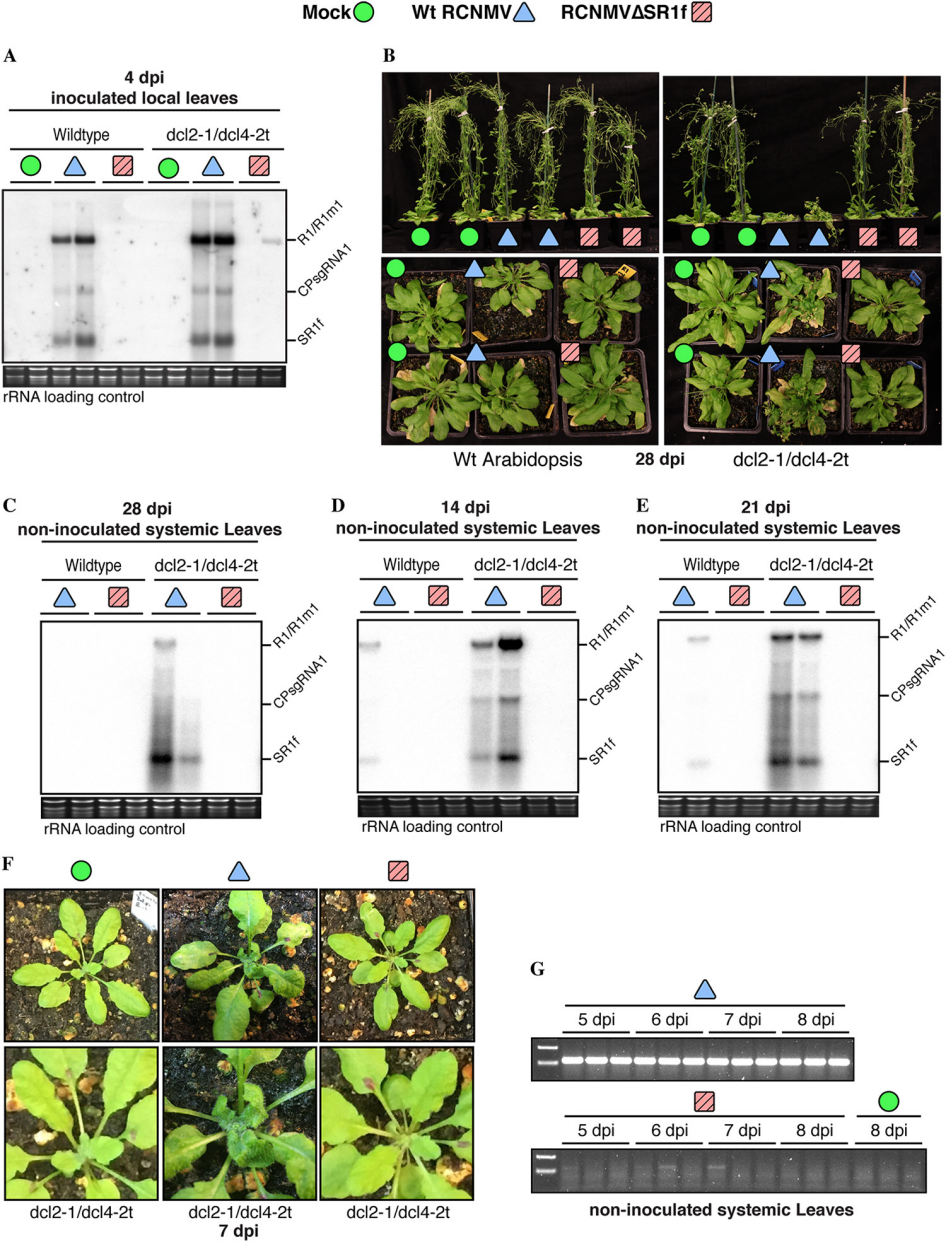
Effect of SR1f knockout on RCNMV infection in *Arabidopsis*. (A) Northern blot from wt-RCNMV-infected and *dcl2-1/dcl4-2t Arabidopsis* plants at 4 dpi. (B) Symptoms in wild-type and *dcl2-1/dcl4-2t Arabidopsis* at 28 dpi. (C) Northern blot from plants shown in panel B at 28 dpi. (D and E) Northern blot from RCNMV-infected wt and *dcl2-1/dcl4-2t Arabidopsis* at 14 dpi (D) and 21 dpi (E). (F) Symptoms in *dcl2-1/dcl4-2t Arabidopsis* at 7 dpi. (G) RT-PCR was performed to verify RNA1 or RNA1-m1 accumulation in plants from panel F. SYBR Safe-stained rRNA was used as a loading control for the Northern blots.

*Arabidopsis* leaves were mechanically inoculated with sap from mock-, wt RCNMV-, and RCNMVΔSR1f-infected N. benthamiana. At 4 dpi, only wt RCNMV replication was detected in the local inoculated leaves of wt and *dcl2-1/dcl4-2t Arabidopsis* (Fig. 4A). By 28 dpi, no symptoms were observed in wt *Arabidopsis* inoculated with either wt RCNMV or RCNMVΔSR1f (Fig. 4B). However, in *dcl2-1/dcl4-2t Arabidopsis*, symptoms such as chlorosis, necrosis, severely mosaic and epinastic leaves, and defective bolting were observed in wt-RCNMV-inoculated plants but not in RCNMVΔSR1f-inoculated plants at 28 dpi (Fig. 4B). Consistent with the symptoms, only wt RCNMV replication was detected in the systemic leaves of only *dcl2-1/dcl4-2t Arabidopsis* (Fig. 4C). In wt *Arabidopsis* at 14 and 21 dpi, wt RCNMV replication was inconsistently detected and RCNMVΔSR1f replication was not detected in any plants. In *dcl2-1/dcl4-2t Arabidopsis* at 14 and 21 dpi, even though we consistently detected the replication of wt RCNMV, we did not detect RCNMVΔSR1f replication (Fig. 4D and E). Similarly, the symptoms always appeared only in wt-RCNMV-inoculated *dcl2-1/dcl4-2t Arabidopsis* at different time points. In other independent experiments, including only *Arabidopsis dcl2-1/dcl4-2t* plants, the symptoms and wt RNA1 accumulation were consistent even at early time points (Fig. 4F). However, RNA1-m1 accumulation was neither consistent nor reproducible in RCNMVΔSR1f-inoculated *dcl2-1/dcl4-2t* plants, as detected by RT-PCR (Fig. 4G).

If SR1f were required only for silencing suppression, the *dcl2-1/dcl4-2t* knockout should rescue the replication of RCNMVΔSR1f to wt RCNMV levels because the virus would not need a silencing suppression function to infect these silencing-deficient plants. However, knocking out the antiviral RNAi system in *Arabidopsis dcl2-1/dcl4-2t* plants did not rescue the replication of RCNMVΔSR1f to wt-RCNMV levels. Therefore, we conclude that SR1f has an essential function for infection other than silencing suppression, although we do not rule out the possibility that SR1f may also play a role in silencing suppression.

**FIG 5:**
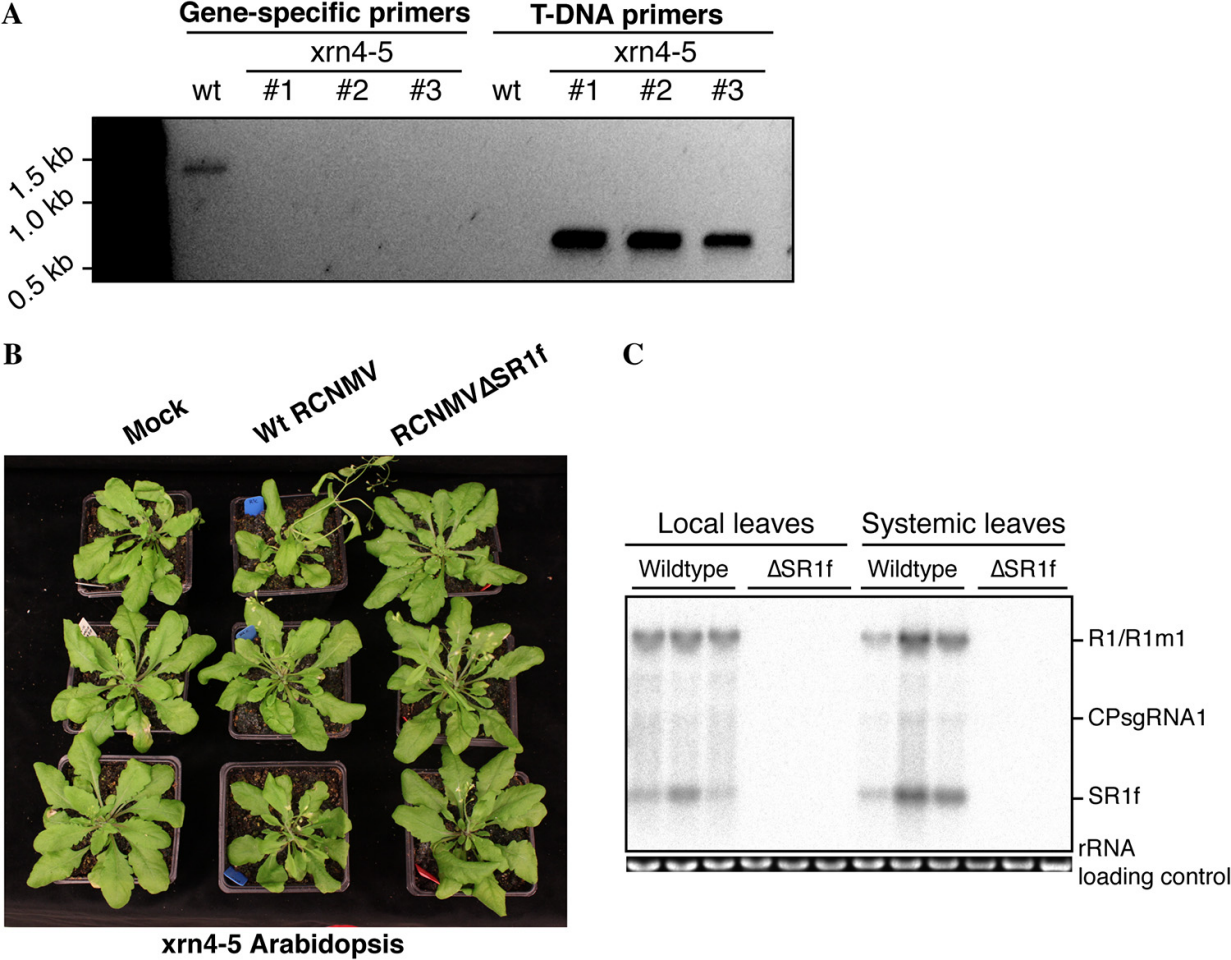
RCNMV infection in *xrn4-5 Arabidopsis*. (A) Genotyping *xrn4-5* mutant *Arabidopsis*. Gene-specific primers refer to the forward and reverse primer for *XRN4* gene. T-DNA primers refer to the pDAP101 vector primer paired with *XRN4* reverse primer. The primer sequences are listed in Table S4 in File 1 in the supplemental material. (B) Symptoms in *xrn4-5 Arabidopsis* at 21 dpi. (C) Northern blot hybridization from plants shown in panel B at 21 dpi.

Next, we sought to determine whether the inability of RCNMVΔSR1f to replicate in *Arabidopsis* is because the 6-base substitution in RNA1-m1 may affect virus replication and/or translation in *cis.* To test this, we inoculated the *xrn4-5* knockout mutant line of *Arabidopsis* with either wt RCNMV or RCNMVΔSR1f. *Arabidopsis xrn4-5* is a loss of function mutant with T-DNA insertion in the *XRN4* gene (52), which was verified by PCR (Fig. 5A). XRN4 is the host cytoplasmic XRN that has been assumed to be responsible for generating xrRNA-derived ncsgRNAs from plant viruses (19, 28, 30, 43). However, the role of XRN4 in generating plant viral xrRNA-derived ncRNA has not been determined *in planta*. If SR1f is generated via exonucleolytic degradation by XRN4, we expect to see no SR1f production in wt-RCNMV-inoculated *xrn4-5* plants. Furthermore, these plants have a functional antiviral RNA silencing machinery. Therefore, if RCNMVΔSR1f did not replicate in wt *Arabidopsis* owing to the lack of SR1f production, we would not expect wt RCNMV replication in *xrn4-5 Arabidopsis*. In contrast, if SR1f is dispensable, and RCNMVΔSR1f did not replicate in wt *Arabidopsis* only because of a *cis*-acting effect of the 6-base substitution in RNA1-m1, we expect wt RCNMV replication to occur in *xrn4-5 Arabidopsis* even without SR1f production. Similar to the observation in wt *Arabidopsis*, there were no discernible symptoms in either wt-RCNMV- or RCNMVΔSR1f-inoculated *xrn4-5* plants (Fig. 5B). Surprisingly, wt RCNMV replication and SR1f accumulation was observed at 21 dpi (Fig. 5C) indicating that XRN4 is not required for generating SR1f. This conclusion supports a report of the BNYVV ncRNA3 accumulation in N. benthamiana plants despite virus-induced gene silencing (VIGS) treatment that reduced *Xrn4* mRNA levels (19). Because SR1f was produced in wt RCNMV-infected *xrn4-5 Arabidopsis*, we were unable to determine whether the lack of RCNMVΔSR1f replication in *Arabidopsis* is because of the absence of SR1f or because of the *cis*-acting effect of the 6-base substitution in RNA1-m1.

**FIG 6:**
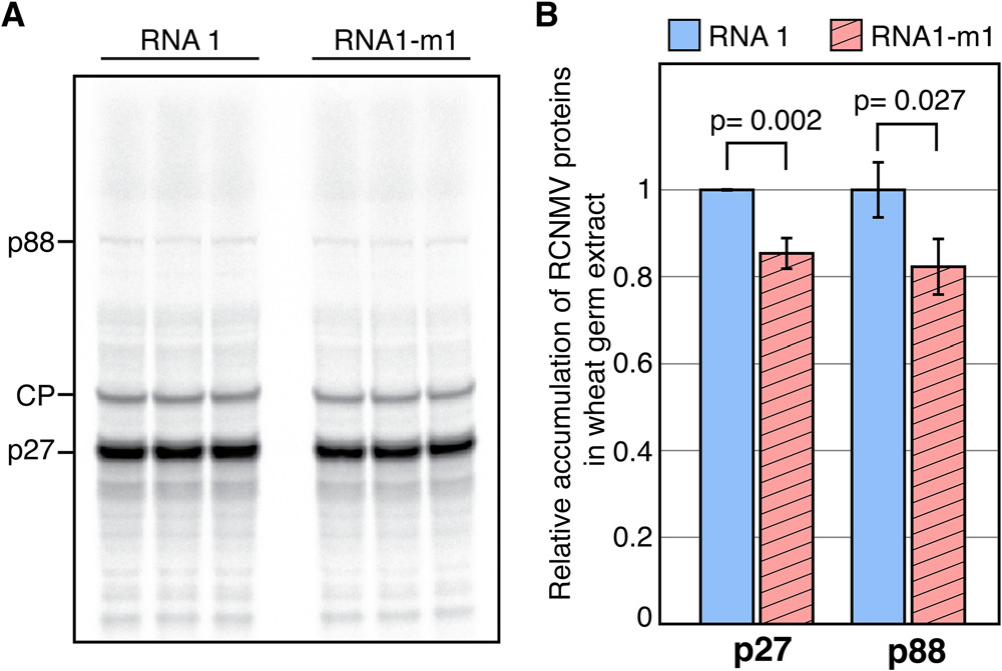
*In vitro* translation of RCNMV RNA1 and RNA1-m1 in wheat germ extract. (A) Polyacrylamide gel electrophoresis of ^35^S-methionine-labeled wheat germ translation products of RCNMV RNA1 and RNA1-m1. Mobilities of viral proteins are indicated on the left. (B) Relative accumulation of RCNMV p27 and p88 in WGE, as determined by measuring the band intensity from panel A and calculating the accumulation of viral proteins relative to those from wt RNA1. Error bars represent the standard deviations (SD).

The 6-base substitution (nucleotides [nt] 3461 to 3466) in the xrRNA structure lies at the 5′ end of the 3′ UTR of RNA1 (Fig. 1B), near the LDFE (nt 3562 to 3566) that is required for translation of RCNMV p88 protein by −1 programmed ribosomal frameshifting (Fig. 1B) (48), and the 3′ TE-DR1 required for cap-independent translation (Fig. 1B) (nt 3596 to 3732) (47). Because of its proximity to these elements, it is possible that the 6-base substitution in RNA1-m1 could affect RNA folding and thus the activity of 3′ TE-DR1 and/or LDFE and thereby suppress RNA1-m1 translation. To test whether the 6-base substitution in RNA1-m1 affects its translation in *cis*, we conducted *in vitro* translation in wheat germ extract. RCNMV p27 and p88 proteins from RNA1-m1 accumulated to about 82 to 85% of the level obtained from wt RCNMV RNA (Fig. 6). This was a statistically significant but minor effect compared to the complete ablation of SR1f RNA and the 4- to 5-fold reduction in virus accumulation in N. benthamiana, caused by this 6-base deletion.

### Effect of infection on host transcriptome.

**FIG 7:**
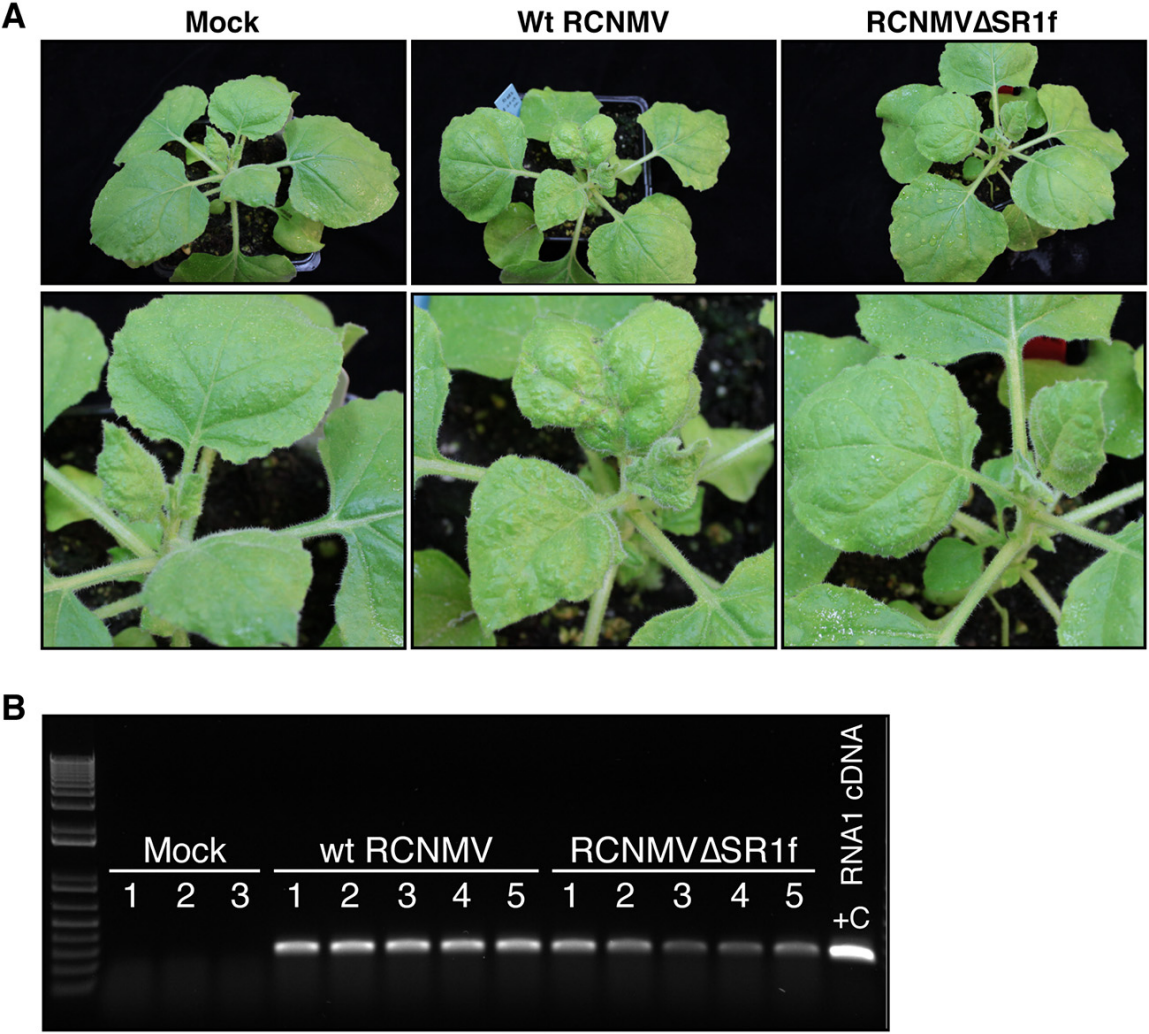
RCNMV-infected N. benthamiana plants used for RNA-seq analysis. (A) Symptoms at 15 dpi. (B) RT-PCR to verify the accumulation RNA1 and RNA1-m1 in wt-RCNMV- and RCNMVΔSR1f-infected plants, respectively. “+C” refers to pR169c (RNA1 infectious clone) positive control.

To understand the role, if any, of SR1f on host gene expression, we performed RNA sequencing (RNA-seq) on plants inoculated with wt RCNMV and RCNMVΔSR1f. Because RCNMVΔSR1f did not replicate in wt *Arabidopsis* and its replication in *dcl2-1/dcl4-2t Arabidopsis* was not consistent and reproducible, we instead performed RNA-seq on RCNMV-infected N. benthamiana. At 15 dpi, necrosis, leaf curling, and mosaic symptoms were observed in wt-RCNMV-inoculated plants, while no symptoms were observed in RCNMVΔSR1f-inoculated plants (Fig. 7A). One noninoculated leaf was collected from mock-, wt-RCNMV-, and RCNMVΔSR1f-inoculated plants at 15 dpi. Both wt RCNMV and RCNMVΔSR1f infections were confirmed by RT-PCR (Fig. 7B). In addition, we performed RT-PCR with RNA1-specific and RNA1-m1-specific primers to ensure there was no cross-contamination among the samples. RNA-seq libraries were prepared using 3, 4, and 4 biological replicates for mock-, wt-RCNMV-, and RCNMVΔSR1f-inoculated plants, respectively. The sequencing data were processed and aligned to (i) N. benthamiana 1.0.1 genome, (ii) RCNMV RNA1 or RNA1-m1, and (iii) RCNMV RNA2. From total host mRNA-mapped reads plus RCNMV-mapped reads, the proportion of reads (means ± the standard deviations [SD]) that mapped to RCNMV genome was ca. 17.4 ± 3.1% in wt-RCNMV-infected plants and ca. 5.2 ± 2.2% in RCNMVΔSR1f-infected plants.

**FIG 8:**
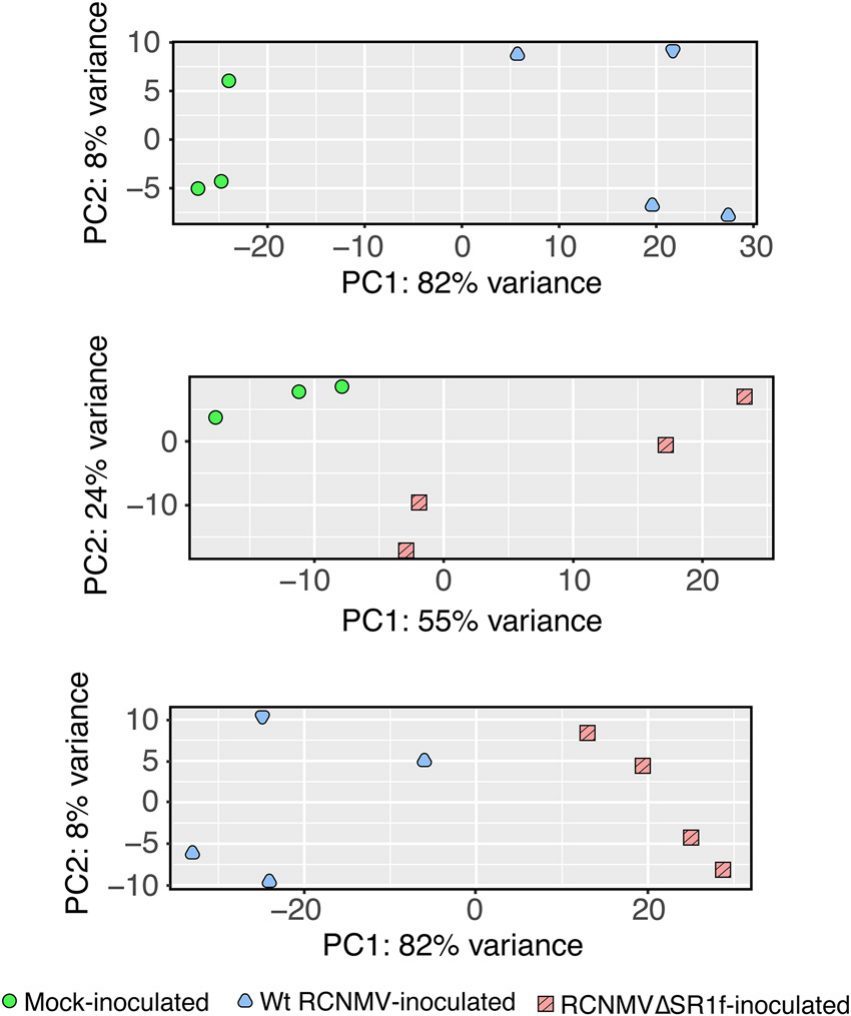
Principal-component analysis (PCA) of RNA-seq data. Regularized log-transformed data from DESeq2 for each of the three comparisons were used to generate the PCA plots.

Next, DESeq2 (53) was used to identify differentially expressed genes (DEGs; output in File 2 in the supplemental material). We compared the results for the wt RCNMV versus mock analyses to those for the RCNMVΔSR1f versus mock analyses to understand how the presence of SR1f affects the host’s transcriptional response to virus infection. Principal-component analysis (PCA) distinguished wt RCNMV infection data from mock-inoculation data and RCNMVΔSR1f infection data with 82% variance (Fig. 8). In contrast, there was only 55% variance between mock-inoculation and RCNMVΔSR1f infection data with two biological replicates of RCNMVΔSR1f-infected plants very close to the mock-inoculated plants (Fig. 8). This shows that the major variation in RNA-seq data among all the samples can be explained by different inocula.

### Differentially expressed genes.

**FIG 9:**
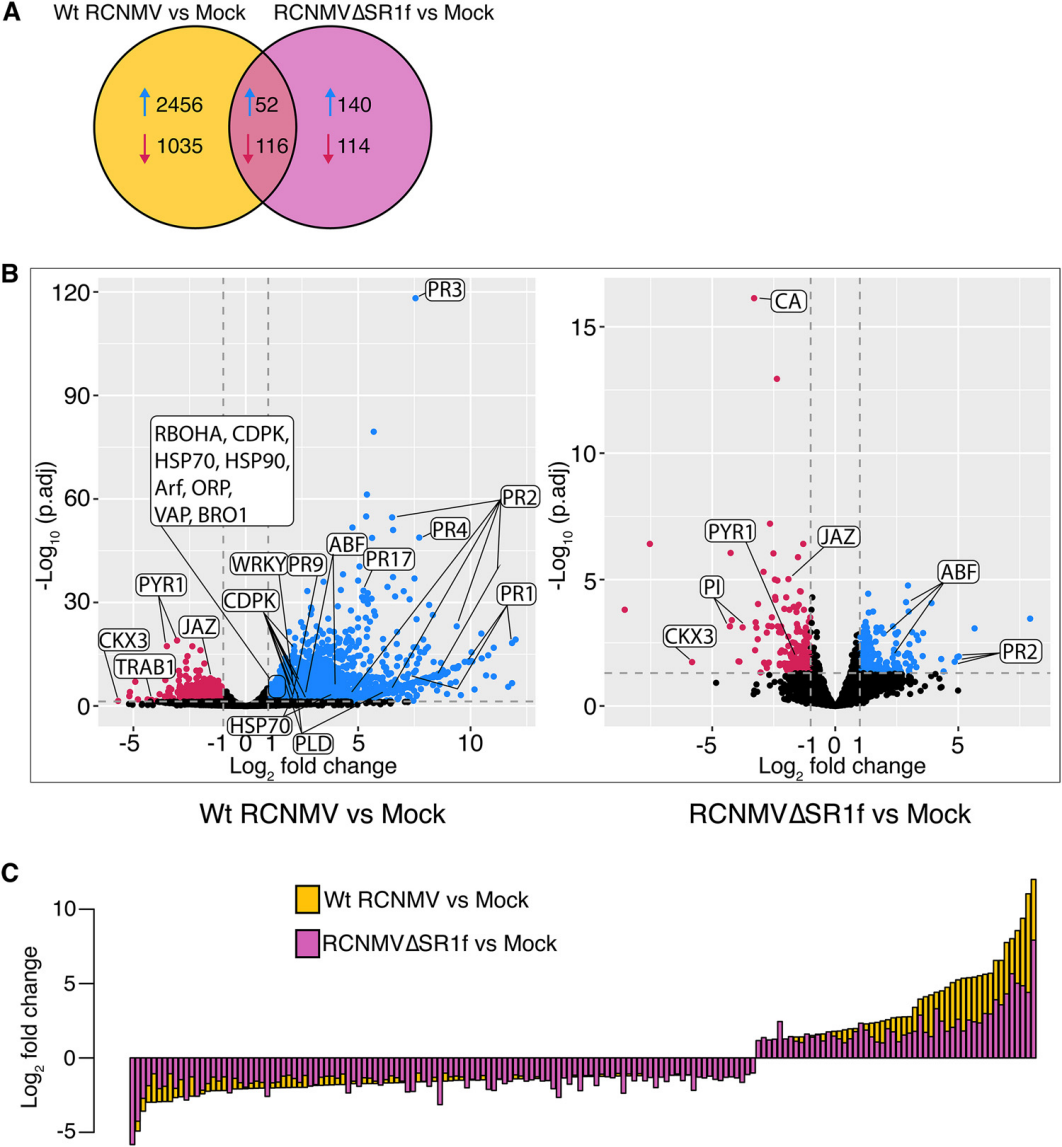
Differentially expressed genes (DEGs) in wt RCNMV versus mock and RCNMVΔSR1f versus mock data sets. (A) Venn diagram showing the number of upregulated and downregulated DEGs. (B) Volcano plots showing log_2_-fold change and adjusted *P* values for all DEGs. Abbreviations: PR, pathogenesis-related protein; WRKY1, WRKY transcription factor; RBOHA, respiratory burst oxidase homolog protein A; ABF, abscisic acid responsive element-binding factor; PYR1, abscisic acid receptor; TRAB1, bZIP transcription factor-ABA signaling; CKX3, cytokinin dehydrogenase; JAZ, jasmonate-zim-domain protein 3; CA, carbonic anhydrase; PI, proteinase inhibitor I-B; CDPK, calcium-dependent protein kinase; HSP, heat shock proteins; Arf, ADP-ribosylation factor; ORP, oxysterol-binding protein-related protein; VAP, vesicle-associated membrane protein-associated protein; BRO1, vacuolar protein-sorting protein bro1; PLD, phospholipase D. (C) Histogram comparing the log_2_-fold change of DEGs that are common in wt RCNMV versus mock and RCNMVΔSR1f versus mock data sets.

We define DEGs as those having an absolute (log_2_-fold change) of >1 and an adjusted *P* value of <0.05. From wt RCNMV versus mock data analysis, we found 3,659 DEGs in which 2,508 genes were upregulated and 1,151 genes were downregulated (Fig. 9A). From RCNMVΔSR1f versus mock data analysis, we found only 422 DEGs in which 192 genes were upregulated and 230 genes were downregulated (Fig. 9A). Consistent with the symptom observation, volcano plots show the greater impact of wt RCNMV infection on N. benthamiana gene expression with greater fold change and more significant results than for RCNMVΔSR1f infection (Fig. 9B). Next, we plotted the 52 upregulated and 116 downregulated DEGs shared among both data sets (Fig. 9C). The shared upregulated genes show a larger fold change (versus mock strains) in wt RCNMV infection than in RCNMVΔSR1f infection, suggesting that these genes are upregulated in response to virus infection although their magnitude of expression can result from both RCNMV replication levels and/or the presence of SR1f *per se*. Interestingly, the shared downregulated genes show a similar fold change in both conditions suggesting that these genes are downregulated in response to RCNMV infection rather than by the presence of SR1f. We hypothesize that SR1f may sequester the 5′→3′ XRN protein(s) disrupting the cellular RNA decay machinery, thus stabilizing both cellular and viral RNAs. Further molecular assays need to be performed to test this hypothesis.

### GO enrichment analysis.

The differentially upregulated and downregulated genes were used separately in wt RCNMV versus mock comparisons and RCNMVΔSR1f versus mock comparisons to identify the enriched GO terms. The upregulated genes in the wt RCNMV versus mock data set enriched 977 GO terms. The top 15 significant terms classified mainly into the biological process category (Table 1). The downregulated genes in the wt RCNMV versus mock data set enriched 501 GO terms, with the top 15 terms classifying into the cellular component category and mainly related to the photosynthesis machinery (Table 1). However, in the RCNMVΔSR1f versus mock data set, the upregulated genes enriched only 37 GO terms, and the downregulated genes enriched 319 GO terms, mainly biological process category for top 15 terms (Table 2).

**TABLE 1:** Top 15 enriched GO terms in wt RCNMV versus mock data sets using upregulated and downregulated differentially expressed genes

GO term	Ontology^*a*^	Input no.^*b*^	Background no.^*c*^	Adjusted *P*
Upregulated DEG dataset				
Response to stimulus	BP	971	5,510	1.57E–153
Response to stress	BP	677	3,196	7.96E–131
Response to chemical	BP	611	2,743	1.38E–124
Cellular anatomical entity	CC	1,997	20,476	1.16E–115
Cell periphery	CC	643	3,773	5.85E–86
Response to oxygen-containing compound	BP	376	1,504	4.44E–84
Plasma membrane	CC	585	3,279	1.04E–83
Cellular process	BP	1,285	11,150	5.01E–83
Binding	MF	1,144	9,385	7.44E–83
Response to organic substance	BP	402	1,760	1.35E–80
Cellular response to chemical stimulus	BP	356	1,412	2.64E–80
Cellular response to stimulus	BP	515	2,738	2.40E–79
Intracellular	CC	1,709	17,437	1.48E–74
Defense response	BP	335	1,361	4.98E–73
Metabolic process	BP	1,173	10,224	2.08E–70
				
Downregulated DEG dataset				
Cellular anatomical entity	CC	884	20,476	3.72E–43
Plastid	CC	318	4,526	9.17E–37
Thylakoid	CC	93	487	1.01E–35
Chloroplast	CC	298	4,164	1.42E–35
Chloroplast thylakoid	CC	84	417	7.98E–34
Plastid thylakoid	CC	84	417	7.98E–34
Chloroplast thylakoid membrane	CC	72	337	2.87E–30
Plastid thylakoid membrane	CC	72	337	2.87E–30
Photosynthetic membrane	CC	73	352	4.86E–30
Thylakoid membrane	CC	72	351	2.16E–29
Intracellular	CC	751	17,437	4.94E–27
Membrane	CC	382	6,756	5.75E–27
Organelle	CC	708	16,128	2.38E–26
Membrane-bounded organelle	CC	700	15,953	1.21E–25
Organelle subcompartment	CC	98	775	1.59E–25

a BP, biological process; CC, cellular component; MF, molecular function.

b The input number is the number of DEGs in our data set annotated to the GO term.

c The background number is the number of genes in the background database annotated to the GO term.

**TABLE 2:** Top 15 enriched GO terms in RCNMVΔSR1f versus mock data sets using upregulated and downregulated differentially expressed genes

GO term	Ontology^*a*^	Input no.^*b*^	Background no.^*c*^	Adjusted *P*
Upregulated DEG dataset				
Cell division	BP	14	347	8.77E–06
Cell cycle	BP	14	500	0.000335
Structural constituent of cell wall	MF	4	19	0.002280
ATPase activity	MF	11	439	0.007688
Cyclin-dependent protein kinase holoenzyme complex	CC	4	42	0.020836
Cyclin-dependent protein serine/threonine kinase regulator activity	MF	4	43	0.020836
Cell cycle process	BP	9	370	0.024266
Glucan endo-1,3-β-d-glucosidase activity	MF	3	19	0.024266
Nucleoside triphosphatase activity	MF	12	668	0.024266
Transferase complex, transferring phosphorus-containing groups	CC	6	161	0.024266
Regulation of cyclin-dependent protein serine/threonine kinase activity	BP	4	56	0.024266
Regulation of cyclin-dependent protein kinase activity	BP	4	56	0.024266
Serine/threonine protein kinase complex	CC	4	59	0.024266
Protein kinase complex	CC	4	60	0.024266
Mitotic cell cycle process	BP	6	172	0.024266
				
Downregulated DEG dataset				
Cellular anatomical entity	CC	165	20,476	1.17E–09
Response to stress	BP	53	3,196	1.99E–09
Response to stimulus	BP	72	5,510	3.79E–09
Cytoplasm	CC	116	12,053	1.32E–08
Response to acid chemical	BP	29	1,161	1.93E–08
Response to chemical	BP	46	2,743	1.93E–08
Organic acid metabolic process	BP	27	1,039	3.41E–08
Response to organic substance	BP	35	1,760	5.57E–08
Small molecule metabolic process	BP	32	1,496	5.57E–08
Response to oxygen-containing compound	BP	32	1,504	5.70E–08
Intracellular	CC	144	17,437	7.72E–08
Cellular process	BP	107	11,150	9.71E–08
Cytosol	CC	45	2,853	1.04E–07
Terpenoid biosynthetic process	BP	11	136	1.24E–07
Oxoacid metabolic process	BP	25	1,032	2.87E–07

a BP, biological process; CC, cellular component; MF, molecular function.

b The input number is the number of DEGs in our data set annotated to the GO term.

c The background number is the number of genes in the background database annotated to the GO term.

Lists of (i) all the enriched GO terms, (ii) the genes involved in the GO term found in our data set, and (iii) the *P* values can be found in File 3 in the supplemental material. Subsequently, the revigo tool was used to reduce the redundancy in the enriched GO term data set and for visualization of the more relevant parent GO terms in “biological process,” “cellular components,” and “molecular function.” The terms that were highlighted in the wt RCNMV versus mock upregulated DEG data set included “response to other organism,” “carbohydrate metabolism,” “vesicle,” “cell wall,” “calcium ion binding,” “protein serine-threonine kinase activity” (see Fig. S1 in File 1 in the supplemental material) and the terms in the wt RCNMV versus mock downregulated DEG data set included “hormone-mediated signaling pathway,” “plant-type cell wall loosening,” “chloroplast thylakoid,” “cell wall,” “water channel activity,” and “kinase activity” (see Fig. S1) among others. However, in the RCNMVΔSR1f versus mock data set, only a few terms were highlighted in upregulated DEG data set whereas several terms were highlighted in the downregulated DEG data set (see Fig. S2 in File 1 in the supplemental material). Even though number of downregulated DEGs in both inoculations are very different, the number of highlighted terms that are affected are similar in both inoculations. In contrast, the upregulated DEGs in wt RCNMV infection affect far greater number of molecular functions, biological processes and cellular pathways than in RCNMVΔSR1f infection. This suggests that the greater impact on host by wt RCNMV infection may be because of the upregulated DEGs, rather than the downregulated DEGs, and the greater proportion of DEGs being upregulated may be due to the presence of SR1f sequestering the XRN, as hypothesized above.

### KEGG pathway enrichment analysis.

The DEGs in wt RCNMV and RCNMVΔSR1f infection were used for KEGG pathway enrichment analysis. *A. thaliana*, *Solanum lycopersicum*, and N. tabacum annotations were used as a background database for the analysis. Because more informative terms were found with *Arabidopsis* background, we focused on those for further analysis. More details for the enriched pathways using the other two background databases can be found in File 4 in the supplemental material, and the DEGs involved in selected KEGG pathways can be found in File 5 in the supplemental material. In the wt RCNMV versus mock data set, 119 pathways were identified and 21 of those were enriched with “plant-pathogen interaction,” “MAPK signaling pathway-plant,” and “plant hormone signal transduction” being the most significant (see File 4 in the supplemental material).

**FIG 10:**
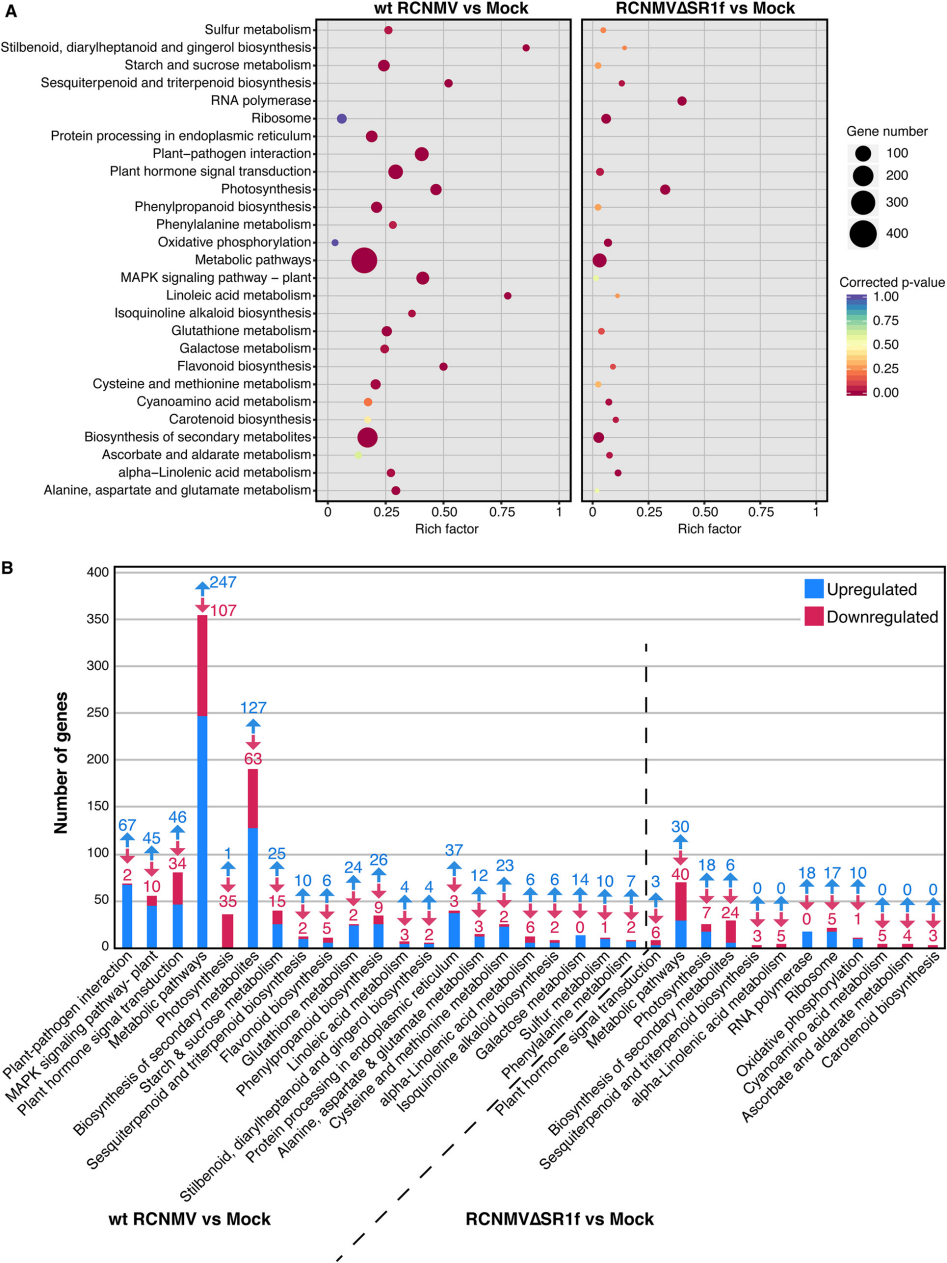
KEGG pathway enrichment analysis. (A) Scatterplot showing the rich factor of the KEGG pathway (*x* axis), number of DEGs present in our data (size of the points), and the corrected *P* values for enrichment (color). Rich factor is the ratio of number of DEGs in the pathway to the total number of genes annotated in the pathway. (B) Histogram showing the number of DEGs for each of the enriched KEGG pathways in wt RCNMV and RCNMVΔSR1f infections. The numbers with the arrows indicate the number of upregulated and downregulated genes in the direction of the arrow.

The rich factor was calculated for each pathway. This is the ratio of number of DEGs in our data set in the pathway to the total number of genes annotated in the pathway in the background database. A rich factor of 1 means all the genes annotated in the pathways are differentially expressed in our data set. In the wt RCNMV versus mock data set, the rich factor of significantly enriched pathways ranged from 0.16 to 0.86 with “stilbenoid, diarylheptanoid, and gingerol biosynthesis,” “linoleic acid metabolism,” “sesquiterpenoid and triterpenoid biosynthesis,” and “flavonoid biosynthesis” pathways having a rich factor greater than 0.5 (Fig. 10A). These pathways are frequently found to be enriched during viral infections (54, 55). Most of the enriched pathways can be grouped under metabolic pathways and it shows the significant impact of wt RCNMV infection on the plant’s metabolism. In contrast, 60 pathways were identified in the RCNMVΔSR1f versus mock data set, and only 12 of those were enriched with “photosynthesis,” “RNA polymerase,” and “metabolic pathways” being the most significant (see File 4 in the supplemental material). None of the enriched pathways have a rich factor greater than 0.5 (Fig. 10A).

In “plant-pathogen interaction” and “MAPK signaling” pathway (enriched only in wt RCNMV infection) most of the DEGs were upregulated (Fig. 10B). These DEGs are involved in both PAMP-triggered immunity and effector-triggered immunity affecting hypersensitive-response (HR) and defense-related gene induction (see Fig. S3 in File 1 in the supplemental material). Transcripts for several calcium-dependent protein kinase (CDPK) genes and one respiratory burst oxidase homolog protein A (RBOHA) gene accumulated at significantly higher levels in wt-RCNMV-infected plants than in mock- or RCNMVΔSR1f-infected plants. This result was not unexpected since plants elicit a reactive oxygen species (ROS) burst as an antiviral defense response, and yet RCNMV requires ROS production for efficient replication (56). It is possible that in wt RCNMV infection, viral RNA and proteins accumulate to a sufficient level to elicit a strong defense response, especially coat protein-mediated HR response (57), and the virus hijacks one of those defense responses (ROS) to accelerate viral replication. This can be seen as high levels of RNA1 accumulation in wt RCNMV infection compared to RNA1-m1 in RCNMVΔSR1f infection in which the host does not elicit a strong defense or ROS response.

Plant-pathogen interactions are greatly impacted by phytohormone signaling (58, 59). The “plant hormone signal transduction” pathway was enriched in both wt RCNMV and RCNMVΔSR1f infections. In this pathway, 80 and 9 genes were differentially regulated during wt RCNMV and RCNMVΔSR1f infection, respectively (Fig. 10B). The salicylic acid pathway, which is key for systemic acquired resistance (SAR) and induction of PR-proteins, was upregulated only in wt RCNMV infection (see Fig. S4 in File 1 in the supplemental material). The abscisic acid pathway was upregulated in both wt RCNMV and RCNMVΔSR1f infection (see Fig. S4 in File 1 in the supplemental material). In addition, in the “photosynthesis” pathway, only 1 gene was upregulated and 35 were downregulated in wt-RCNMV-infected plants, whereas 18 genes were upregulated, and 7 were downregulated in RCNMVΔSR1f infection (Fig. 10B; see also File 5 in the supplemental material). Essentially, 19 components of photosystems I and II, cytochrome *b*_6_/*f* complex, photosynthetic electron transport, and F-type ATPase were mainly downregulated in wt RCNMV infection, while in RCNMVΔSR1f infection, only 1 component of photosystem II was downregulated, and 5 components of photosystems I and II and F-type ATPase were upregulated (see Fig. S5 in File 1 in the supplemental material). In addition to the dysregulated metabolic pathways, a disruption in photosynthesis machinery may contribute to the symptom development in wt RCNMV infection. In the “metabolic pathways,” 247 genes were upregulated, and 107 genes were downregulated in wt-RCNMV-infected plants, while only 30 genes were upregulated, and 40 genes were downregulated in RCNMVΔSR1f-infected plants (Fig. 10B). In the “protein processing in endoplasmic reticulum” pathway, enriched only in wt RCNMV infection, 37 genes were upregulated, while only 3 were downregulated (Fig. 10B). This may be a consequence of endoplasmic reticulum (ER) membrane remodeling during RCNMV replication (60, 61). It would be interesting to test whether RCNMV-infected cells undergo ER stress and elicit an unfolded protein response.

### LRR-RLKs/RLPs and PR genes.

Leucine-rich repeat receptor-like kinases and proteins (LRR-RLKs/RLPs) play important roles in plant development, immunity, and stress responses (62–65). These are the primary components that recognize the pathogen/damage-associated molecular patterns (P/DAMPs) and elicit pattern-triggered immunity (PTI). Of the 317 LRR-RLKs and 86 LRR-RLPs identified in N. benthamiana (66), 38 LRR-RLKs and 15 LRR-RLPs were differentially regulated in wt RCNMV infection (see Table S1 in File 1 in the supplemental material). However, none of the LRR-RLKs/RLPs were differentially regulated in RCNMVΔSR1f infection. Another component of the plant’s innate immune response includes induction of pathogenesis-related (PR) proteins upon pathogen attack. Of 29 PR genes identified in N. benthamiana (67), 12 genes encoding PR-1, PR-2, PR-3, PR-4, PR-9, and PR-11 proteins were differentially upregulated in wt RCNMV infection, whereas only 3 genes, all encoding the PR-2 protein, were upregulated in RCNMVΔSR1f infection (Table 3).

**TABLE 3:** PR genes in N. benthamiana obtained from Li et al. (67) with a log_2_-fold change and adjusted *P* values from RNA-seq analysis using DeSeq2^*a*^

PR family	Protein properties	Unigene ID	wt RCNMV vs mock		RCNMVΔSR1f vs mock	
			Log_2_FC	Adj. *P*	Log_2_FC	Adj. *P*
PR 1	Cysteine-rich secretory protein, allergen V5/Tpx-1-related	Niben101Scf13926g01014				
		Niben101Scf03376g03004	9.92	1.90E–13		
		Niben101Scf00107g03008	11.85	5.73E–19		
		Niben101Scf01999g07002	7.22	5.61E–10		
PR 2	Glucan endo-1,3-β-glucosidase-like, Glycoside hydrolase, family 17	Niben101Scf01001g00005	6.47	4.20E–05		
		Niben101Scf01001g00004			4.96	0.0116
		Niben101Scf01001g00003	8.56	4.82E–10	5.02	0.0110
		Niben101Ctg13736g00004	9.38	8.42E–24	4.85	0.0179
		Niben101Scf04869g03002	6.51	2.19E–55		
		Niben101Scf01001g00006	4.74	0.00035		
PR 3	Chitinase 8, glycoside hydrolase, family 19	Niben101Scf02041g00002	7.55	6.61E–119		
PR 4	Thaumatin-like protein	Niben101Scf01400g00014	7.51	1.13E–37		
		Niben101Scf03436g01016				
PR 5	Pathogenesis-related thaumatin superfamily protein	Niben101Scf00126g00008				
		Niben101Scf05554g05006				
PR 6	Cysteine-rich secretory protein, allergen V5/Tpx-1-related	Niben101Scf00953g05001				
		Niben101Scf04053g01004				
PR 9	Peroxidase 53, heme peroxidase	Niben101Scf03460g04004	1.94	0.0046		
		Niben101Scf07182g05012				
PR 10	Major pollen allergen Bet v 1-M/N, Bet v I type allergen	Niben101Scf03526g00006				
		Niben101Scf10735g00016				
		Niben101Scf02474g01024				
		Niben101Scf01938g04007				
PR 11	Chitinase-3-like protein 2, Glycoside hydrolase superfamily	Niben101Scf06295g04023				
		Niben101Scf01789g04010				
PR 17	Plant basic secretory protein family protein, uncharacterized protein family	Niben101Scf03385g02011	5.24	3.88E–34		
		Niben101Scf03385g01006				
		Niben101Scf01341g01002				
		Niben101Ctg10643g00004				

a FC, fold change; Adj. *P*, corrected *P* value.

### Validation of RNA-seq analysis using qRT-PCR.

**FIG 11:**
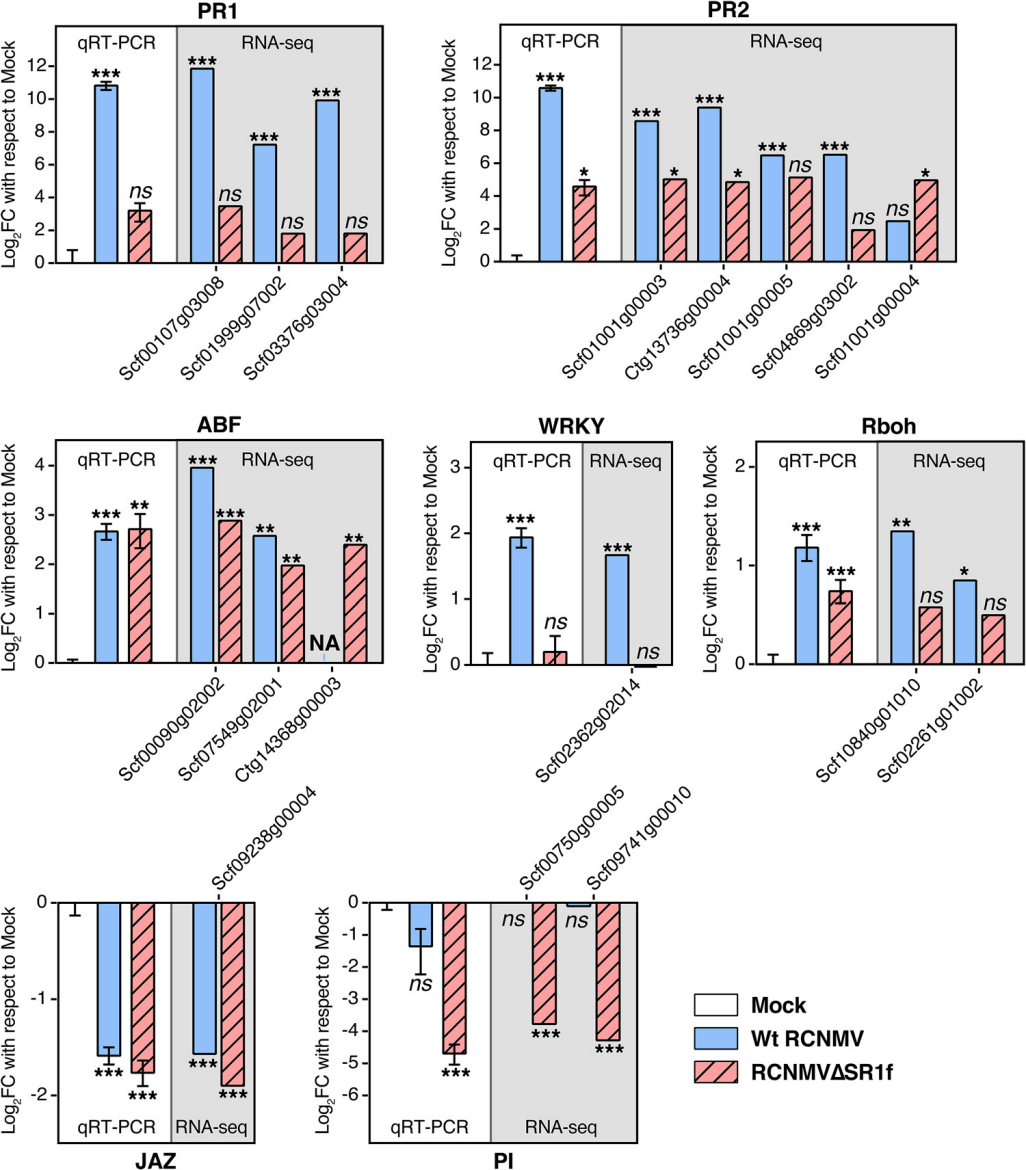
Validation of RNA-seq data by qRT-PCR of candidate genes. NbPP2A and NbL23 were used as reference genes for normalization of qRT-PCR data. Expression values are shown with respect to mock values. RNA-seq data for each gene with one or more Unigene IDs are shown that could be amplified by qRT-PCR (see methods). The Unigene IDs are preceded by “Niben101.” Abbreviations: FC, fold change; PR, pathogenesis-related protein; ABF, abscisic acid responsive element-binding factor; WRKY, probable WRKY transcription factor 33; Rboh, respiratory burst oxidase homolog protein A; JAZ, jasmonate-zim-domain protein 3; PI, proteinase inhibitor I-B. *, *P* < 0.05; **, *P* < 0.01; ***, *P* < 0.001; ns, not significant. Error bars indicate the SEM.

To validate our RNA-seq data analysis, we selected seven host DEGs that were involved in the enriched pathways and quantified their abundance using qRT-PCR. Candidate genes included PR-1, PR-2, abscisic acid responsive element binding factor (ABF), WRKY transcription factor (WRKY), respiratory burst oxidase homolog protein A (Rboh), jasmonate-zim-domain protein (JAZ), and proteinase inhibitor I-B (PI). The log_2_-fold changes and their statistical significance values were consistent between the RNA-seq and qRT-PCR analyses (Fig. 11).

### Read coverage on viral RNAs.

**FIG 12:**
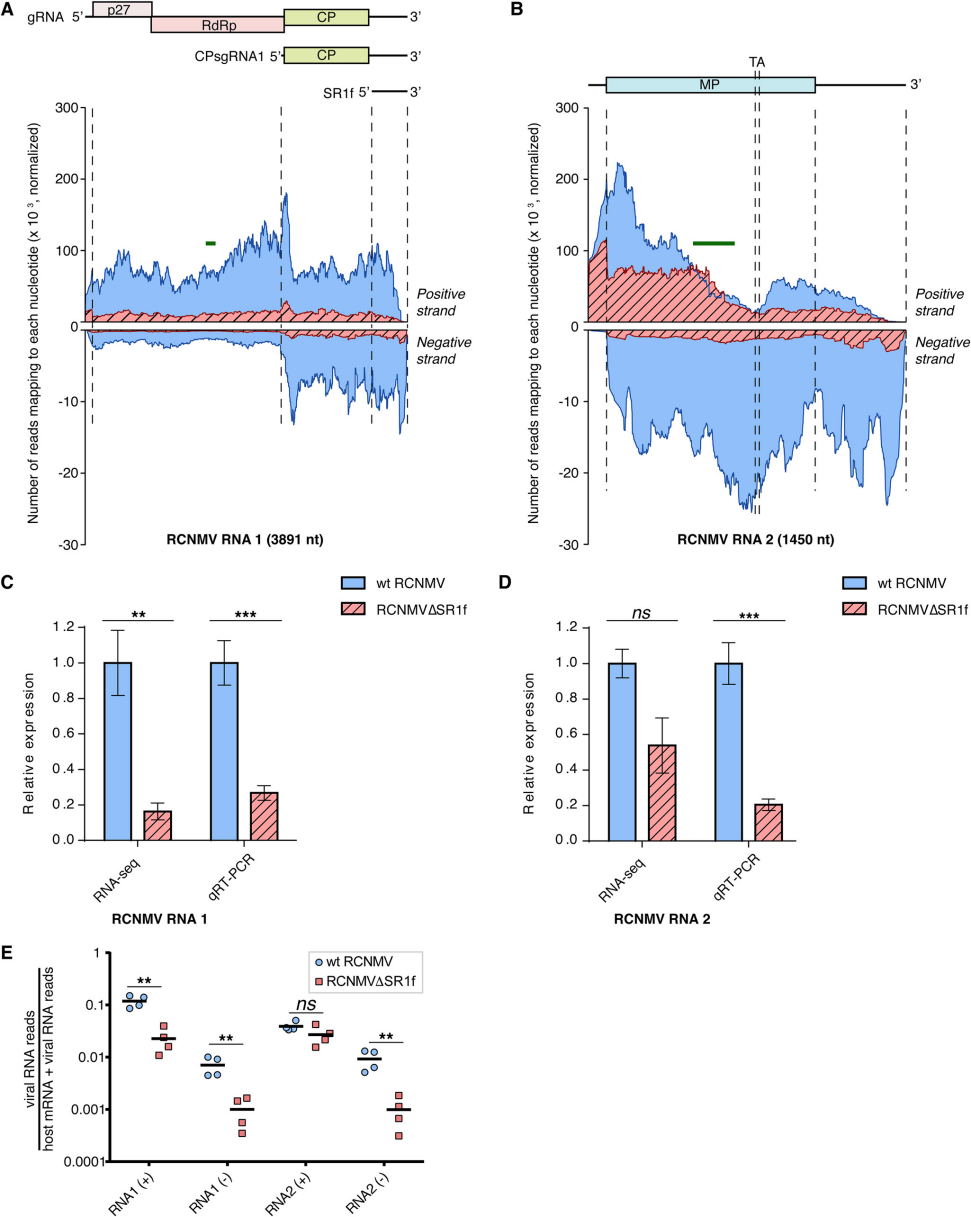
RNA-seq reads mapping to the RCNMV genome. (A) Coverage plots showing the reads mapping to RCNMV RNA1 positive and negative strands. (B) Coverage plots showing the reads mapping to RCNMV RNA2 positive and negative strands. The RCNMV genome organization is shown above the coverage plots. The green bars denote the locations of qRT-PCR amplicon. Note that the *y*-axis scales used for positive- and negative-strand coverage differ by a factor of 10. (C) Relative accumulation of RNA1 (both positive and negative strand) in wt-RCNMV- and RCNMVΔSR1f-infected plants, as measured by RNA-seq and qRT-PCR. (D) Relative accumulation of RNA2 (both positive and negative strand) in wt-RCNMV- and RCNMVΔSR1f-infected plants, as measured by RNA-seq and qRT-PCR. (E) Proportions of viral RNA reads relative to the total number of host mRNA reads plus viral RNA reads. NbPP2A and NbL23 were used as reference genes for normalization of qRT-PCR data. DESeq2-derived scaling factors were used for normalizing RNA-seq data. Adjusted *P* values are indicated by asterisks: *, <0.05; **, <0.01; and ***, <0.001. Error bars indicate the SEM.

**FIG 13:**
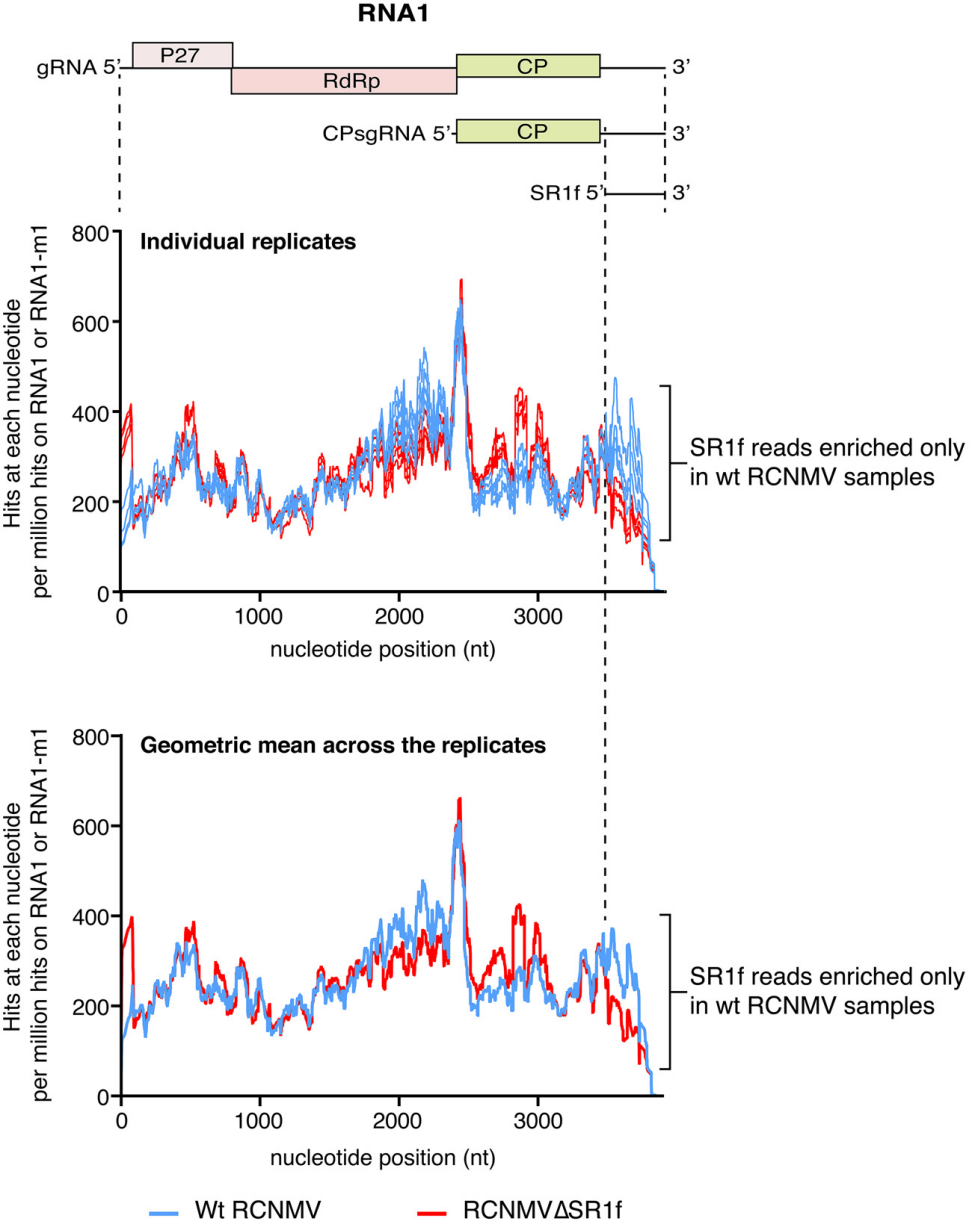
RNA-seq reads mapping to each nucleotide of RCNMV RNA1 and RNA1-m1 normalized to the total number of hits on RNA1 and RNA1-m1, respectively.

RNA-seq reads were mapped to RCNMV RNA1 and RNA2, and the numbers of reads at every nucleotide position on the viral RNA were counted and scaled according to the DESeq2 scaling factor (Fig. 12A and B). Overall, more reads mapped to RNA1 in wt-RCNMV-infected plants than RNA1-m1 in RCNMVΔSR1f-infected plants (Fig. 12A). This was verified using qRT-PCR (Fig. 12C). Read coverage across the negative strand of RNA1 or RNA1-m1 was >10-fold less than the positive strand for most of the genome, as expected. Reads mapping to negative strand clearly show that CPsgRNA1 negative strand was more abundant than RNA1 negative strand upstream of the region corresponding to CPsgRNA1. However, we did not see a particularly high number of reads mapping to the SR1f region. The number of reads at the 5′ ends of the genomic and subgenomic RNAs may be artificially reduced owing to the library preparation kit that we used, which may explain this unexpected observation. However, when the coverage over each nucleotide of RNA1/RNA1-m1 was normalized according to the total number of mapping hits to RNA1 or RNA1-m1, the accumulation of SR1f can clearly be seen only in wt-RCNMV-infected samples (Fig. 13).

The read coverage profile on RNA2 positive strand was not much different in either infection (Fig. 12B). The number of reads mapping to RNA2 in both cases differed by only ∼50%, but the difference was not statistically significant (Fig. 12D). This did not agree with our qRT-PCR validation, where the RNA2 abundance in RCNMVΔSR1f infection was ∼20% of that in wt-RCNMV infection and highly significant, similar to the decrease in RNA1 abundance. In addition, the coverage profile on RNA2 positive strand was rather unexpected. The reads were highly represented in the 5′ half of RNA2; they decline near the middle of the RNA2 sequence and then increase again toward the 3′ end (Fig. 12B). It is unlikely that this is a library preparation artifact because we observed a similar pattern of RNA2 coverage in RCNMV-infected *Arabidopsis* in an independent RNA-seq experiment that used a different library preparation strategy, library preparation kit, and sequencing read length (unpublished data). Interestingly, this inflection point coincides with the transactivator (TA) sequence in RNA2. Intermolecular base pairing between the TA sequence in RNA2 and the 8-nt sequence upstream of the coat protein subgenomic promoter on RNA1 is required to produce negative strand of CPsgRNA1 via premature transcription termination of negative-strand synthesis to generate the template for CPsgRNA1 synthesis (46, 68). The coverage profile of reads on the negative-strand RNA1 or RNA1-m1 showing a greater number of reads in the CPsgRNA1 region further supports the premature transcription termination model (Fig. 12A).

The proportion of individual viral RNAs relative to the total number of host mRNA reads revealed that positive- and negative-strand RNA1 and negative-strand RNA2 are more abundant in wt-RCNMV-infected plants than in RCNMVΔSR1f-infected plants (Fig. 12E). However, there is no significant difference in abundance of positive-strand RNA2 in either infection (Fig. 12E). Upon inspecting Fig. 12E more closely, we observed that the proportion of reads from positive-strand RNA2 is less than positive-strand RNA1 in wt-RCNMV-infected plants (Fig. 12E). In contrast, the proportion of reads from negative-strand RNA1 is similar to negative-strand RNA2 in wt-RCNMV-infected plants, and the proportion of reads from positive- and negative-strand RNA1 is similar to that for positive- and negative-strand RNA2, respectively, in RCNMVΔSR1f-infected plants (Fig. 12E). This leads us to speculate that SR1f may negatively regulate the synthesis of positive-strand RNA2 in late stages of infection.

## DISCUSSION

In this study, we assessed the role of the noncoding RNA of an RNA virus interacting with its host using N. benthamiana and *Arabidopsis* as a model system. We also provide a comparative genome-wide transcriptomic analysis in N. benthamiana to assess how RCNMV infection and the presence of SR1f affect cellular gene expression.

### Lack of SR1f reduces virus levels and symptoms.

Reduced symptoms and decreased viral RNA accumulation in N. benthamiana and *Arabidopsis* plants inoculated with SR1f-deficient RCNMV mutant suggests an important role for SR1f in establishing a successful infection (Fig. 2 and 4). Notable differences in symptoms in N. benthamiana infected with wt RCNMV and RCNMVΔSR1f (Fig. 2B and C) contrasts with a previous report (16), which reported (but did not show) mosaic symptoms in systemic leaves in N. benthamiana infected with several SR1f-deficient RCNMV RNA1 mutants, including the mutant, RNA1-m1, that we have used in our study. However, similar to our observation, it has been shown that a Tobacco necrosis virus (TNV)-D mutant, that cannot make the ncsgRNA, produced milder symptoms than the wt TNV-D infection in N. benthamiana (28). The authors of that study also showed that viral RNA accumulation was reduced in TNV-D mutant infection compared to the wt TNV-D infection, which is similar to the previous RCNMV report (16) and our results (Fig. 2E and Fig. 12C and D). Thus, throughout this study, we cannot distinguish whether most differences in host response were due to SR1f *per se* or due to reduced virus load in general.

### Defective antiviral RNA silencing pathway does not rescue RCNMVΔSR1f replication.

Based on the previous reports that have demonstrated the RNA silencing suppressor activity of ncsgRNAs of WNV, DENV, Kunjin virus (KUNV), and BNYVV (37, 38, 43), we hypothesized that RCNMV SR1f may function as an RNA silencing suppressor. If this were the only function of SR1f, we would expect more symptoms and viral RNA accumulation in RCNMVΔSR1f-inoculated *dcl2-1/dcl4-2t Arabidopsis* plants than in wt *Arabidopsis* because this mutant lacks the antiviral RNA silencing machinery. However, *Arabidopsis dcl2-1/dcl4-2t* mutant did not rescue the replication of RCNMVΔSR1f (Fig. 4). Furthermore, previous reports have shown that the formation of the 480-kDa RCNMV replication complex and the subsequent replication of RNA1 or RNA2 alone is sufficient to suppress RNA silencing in N. benthamiana (69), and the movement protein encoded by RNA2 is also a suppressor of RNA silencing (70). Therefore, we concluded that SR1f performs an essential function other than RNA silencing suppression. This does not rule out the possibility that it may also play a role in RNA silencing suppression, for example, similar to the BNYVV ncRNA3 that complements BNYVV p14 protein to suppress RNA silencing (43).

### XRN4 is not required to generate SR1f.

A key question in plants is which XRN is responsible for generating ncsgRNAs via blockage at the xrRNA structure? In plants, XRN4 is the only known cytoplasmic 5′→3′ XRN, a functional equivalent of XRN1 in yeast and mammals and has been assumed to be responsible for generating xrRNA-derived ncsgRNAs (19, 28, 30, 43). A previous report showed that ectopic expression of plant XRN4 in Saccharomyces cerevisiae (*Δxrn1* background) could generate BNYVV ncRNA3 (19). The authors of that study also demonstrated that VIGS knockdown of XRN4 affects BNYVV accumulation and inhibits its systemic spread in N. benthamiana; however, BNYVV ncRNA3 was still present (19), suggesting that XRN4 may not be necessary for ncRNA3 biogenesis, although residual XRN4 may be present in VIGS knockdown plants. In our experiment, we detected high levels of SR1f in wt-RCNMV-infected *xrn4-5 Arabidopsis* plants (Fig. 5) that have a T-DNA insertion in exon 18 of the only *XRN4* gene, so they do not make a functional XRN4 (52), thereby showing the lack of requirement of XRN4 to produce SR1f. Thus, if XRN4 can generate xrRNA, it is not the only exoribonuclease that does so. In fact, diverse 5′→3′ exoribonucleases, unrelated to XRN1, such as yeast Dxo1 and bacterial RNase J1, have been shown to be capable of generating SR1f *in vitro* (27). Even flaviviral xrRNAs can block different 5′→3′ exoribonucleases (25). This raises important questions, such as which exonuclease(s) are responsible for the production of xrRNA-derived ncsgRNAs *in planta* during infection? Is there a yet-unidentified cytoplasmic 5′→3′ XRN in plants that is responsible for making viral ncsgRNAs? Can other plant XRNs functionally replace XRN4 in its absence? For characterization of xrRNA-derived ncsgRNAs of plant viruses, one could screen and identify *Arabidopsis* 5′→3′ exoribonuclease loss-of-function mutants that cannot make viral ncsgRNAs.

### Effects of wt RCNMV and RCNMVΔSR1f on the host transcriptome.

To test how the cellular gene expression is affected in N. benthamiana infected with wt RCNMV and RCNMVΔSR1f, we conducted RNA-seq. Based on read counts, wt RCNMV accumulated five times as much as the RCNMVΔSR1f mutant, so we cannot distinguish effects of loss of SR1f *per se* from those due to less virus accumulation in general. However, in the plants infected with wt RCNMV, we can identify host genes that may be affected by presence of SR1f. According to our RNA-seq data, almost two-thirds of DEGs are upregulated (69%) in wt RCNMV infection, whereas in RCNMVΔSR1f infection, an almost equal proportion of DEGs are upregulated (45%) and downregulated (55%) (Fig. 9A). This pattern of a greater proportion of DEGs being upregulated (i.e., show an increased level of mRNA) in a virus infection that produces an xrRNA-derived ncsgRNA has been reported previously in *Flavivirus*-infected cells (42). In that report, the amount of XRN1 available for normal turnover of mRNAs was found to be reduced in infected cells, owing to its tight association with flaviviral sfRNA, thus sequestering it from its normal cellular activities, resulting in abnormal stabilization of cellular mRNAs. In their experiment, the wild-type KUNV and the sfRNA-mutant KUNV replicated to the same level, and these researchers were therefore able to attribute their results to the presence of the sfRNAs (42). However, in our case, wt RCNMV replicates to a higher level than RCNMVΔSR1f, and the greater proportion of DEGs being upregulated may therefore be due to increased cellular transcriptional response to virus replication, in addition to any increased mRNA stability arising from disruption of RNA decay pathway by sequestration of a 5′→3′ XRN by SR1f. Even though it has not been shown that RCNMV SR1f can stabilize cellular RNAs *in vivo*, it can increase RCNMV RNA2 accumulation in BY-2 cell-free replication assays (16). According to the GO term and KEGG pathway enrichment analysis, wt RCNMV infection disrupts more cellular pathways and to a greater extent than RCNMVΔSR1f infection (Fig. 10; see Fig. S1 to S5 in File 1 in the supplemental material). Which of these effects can be attributed to host response to virus infection in general and which are specifically due to presence of SR1f remains to be determined. Either way, the large reduction in virus accumulation due to absence of SR1f demonstrates that SR1f plays an important role in the virus life cycle.

We also looked at the differential expression of a few cellular genes in our data set that are known to be coopted by RCNMV and other tombusvirids, such as tomato bushy stunt virus (TBSV) and carnation Italian ringspot virus (CIRV). DEGs in the enriched pathways that are known to be recruited by RCNMV included Rboh and CDPKs (56). In addition to these, genes encoding phospholipase D (PLD), heat shock protein 70 (HSP70), HSP90, ADP-ribosylation factor (Arf) were also upregulated only in wt-RCNMV-infected plants (Fig. 9B). PLD enzymes catalyze the production of phosphatidic acid, which interacts with RCNMV replication protein p27 and promotes RCNMV replication (71). HSP70 and HSP90 interact with RCNMV p27 at the ER membrane, and this interaction is required for the formation of a functional 480 kDa RCNMV replicase complex for successful RCNMV replication (72). RCNMV replication protein p27 also interacts with Arf1, which is a ubiquitous, highly conserved GTPase involved in the formation of COPI vesicles on Golgi membranes, and relocalizes it from the Golgi apparatus to the RCNMV replication site on the ER (61). Selected DEGs that are recruited by other tombusvirids include oxysterol-binding protein-related protein (ORP), vesicle-associated membrane protein-associated protein (VAP), and vacuolar protein-sorting protein bro1 (BRO1), all of which were upregulated only in wt-RCNMV-infected plants (Fig. 9B). ORP and VAP are coopted by TBSV and CIRV for redistribution of sterols to the virus replication sites (73). BRO1 is required for efficient TBSV replication by recruiting ESCRT-III factors in the virus replication complexes (74). The list of Unigene IDs of the above-mentioned genes, and data on their differential expression can be found in Table S2 in File 1 in the supplemental material. In summary, RCNMV induces expression of several proteins shown to be coopted by TBSV or CIRV, while also inducing and using different proteins. This reveals the similarities and differences among viruses in different genera of the *Tombusviridae*.

### Effect of RCNMVΔSR1f on viral RNA levels.

RNA-seq reads mapped to RCNMV RNA1 and RNA2 showed some expected features such as greater abundance of reads mapping to positive strand than the negative strand. The greater number of reads mapping to CPsgRNA1 sequence in the negative strand (Fig. 12A and B) compared to the rest of RNA1, rather than a uniform distribution of reads along the full-length negative strand of RNA1, supports the premature transcription termination model for the synthesis of negative-strand CPsgRNA1 (46). According to this model, CPsgRNA1 positive strand is transcribed from the 3′ terminus of incomplete RNA1 negative strand that arises as a result of premature transcription termination during the synthesis of negative-strand RNA1 (46).

Corresponding to the accumulation of CPsgRNA1 and SR1f subgenomic RNAs, we expected to see more reads in the positive strand in these regions compared to the upstream region of RNA1. However, we saw more reads only for the CPsgRNA1 region, which includes SR1f sequence, but no additional increase in reads was observed for SR1f-specific sequence. We think the lack of an even higher number of reads corresponding to the SR1f portion of RNA1 may be an artifact from the library preparation (Fig. 12A). The library preparation kit that we used utilizes random hexamer-mediated cDNA synthesis as its first step, followed by bead clean-ups that retained reads averaging ∼300 nt. Thus, cDNAs from regions within 300 nt of the 3′ end would be <300 bp and would have been removed, and we would therefore see fewer reads than expected mapping to this region (Fig. 12A). In contrast, the reads mapping to CPsgRNA1 would be mostly retained. However, most of the cDNAs synthesized from CPsgRNA1 would terminate at its 5′ end, and therefore only the 5′ end of CPsgRNA1 would be over-represented in our sequencing data. Indeed, we observe this phenomenon at the 5′ end of positive-strand RNA1, CPsgRNA1, and RNA2 and the coterminal 5′ ends of the negative-strand RNA1 and CPsgRNA1 (Fig. 12A and B). Despite this limitation, the accumulation of SR1f can clearly be seen only in wt-RCNMV-infected samples when the RNA1/RNA1-m1 coverage was normalized to the total number of mapping hits to RNA1 or RNA1-m1 (Fig. 13).

### Possible functions of SR1f.

In summary, using RCNMV SR1f as a model to study xrRNA-derived ncsgRNAs of plant viruses, we show that SR1f plays a key role in virus accumulation and symptom development, possibly by regulating virus and host gene expression and counteracting plant’s defense responses. We conclude that the primary function of SR1f is not an RNA silencing suppressor but may have an important role in counteracting plant defenses and/or modulating virus life cycle by a as-yet-unknown mechanism. Using a reporter system, it has been shown that RCNMV SR1f *trans*-inhibits both cap-independent and cap-poly(A)-dependent translation *in vitro* and *in vivo* in BY-2 protoplast, and it suppresses cap-poly(A)-dependent translation more efficiently than it inhibits 3′ TE-DR1-mediated (viral) cap-independent translation (16). Therefore, the authors hypothesized that accumulation of SR1f may sequester translation initiation factors and/or ribosomal small subunits and suppress translation of host’s cellular mRNAs. However, how RCNMV-infection and SR1f accumulation in plants affects cellular mRNA translation has not been studied yet.

SR1f may also regulate translation of viral RNAs, as has been shown for the sgRNA2 of BYDV, which, like SR1f, contains the BTE. Via its BTE, which binds translation factor eIF4G, sgRNA2 selectively inhibits translation of genomic RNA relative to that of the subgenomic RNA that encodes movement and coat proteins (75). As hypothesized previously (16), SR1f may do the same, but favor translation of CPsgRNA1 and RNA2. Thus, absence of SR1f would perturb optimal regulation of translation of viral RNAs by viral RNA.

It has been shown that RCNMV SR1f and TNV-D svRNA are packaged in the virions (16, 28), unlike WNV sfRNA (15). This suggests that SR1f may also have a role in the early stages of infection. Based on previous reports, results presented in this study and some of our unpublished data, we present the following hypothesis. In the early stages of infection when the specific antiviral pathways have not been triggered, a cytoplasmic 5′→3′ XRN could degrade RCNMV genomic RNAs, which (unlike flavivirus RNAs) are uncapped. However, the copackaged SR1f could sequester the XRN, thus minimizing degradation of viral genomic RNA, allowing it to initiate translation followed by replication. Having an uncapped genome may be the selective pressure resulting in the packaging of SR1f. TNV-D has an uncapped genome and its svRNA is also packaged (28). BNYVV genomic RNAs are capped, but we are unaware of any evidence indicating whether its ncRNA3 is packaged. In contrast to tombusvirids, the cap on flaviviral RNA may provide initial protection or delay in viral RNA degradation (thus explaining the absence of packaged sfRNA [15]), that in RCNMV and TNV-D may be provided by the SR1f and svRNA, respectively, which are present immediately upon RNA entry from the virion. This initial assistance from SR1f would minimize the degradation of viral genomic RNAs that can kick-start the production of the replicase proteins, followed by viral RNA replication. Accumulation of viral RNAs and proteins will elicit antiviral defense responses, including the ROS burst, which is hijacked by RCNMV to accelerate its replication efficiency (56). At a later stage, SR1f accumulation may inhibit translation of viral RNAs by binding and sequestering eIF4F, as hypothesized before (16), making them available for encapsidation similar to the riboregulator function of BYDV sgRNA2 (75, 76). On the other hand, RCNMVΔSR1f does not produce SR1f. Therefore, 5′→3′ XRN may quickly degrade viral genomic RNAs, thus reducing accumulation of viral products. Moreover, the plant defense responses will not be triggered, and there will be no ROS burst that RCNMV exploits for an efficient replication.

## MATERIALS AND METHODS

### *In vitro* transcription of RCNMV RNAs.

RCNMV plasmid constructs used for *in vitro* transcription were described previously (49). pRC169c and pRC2|G are cDNA clones with T7 promoter for *in vitro* transcription of infectious RCNMV RNA1 and RNA2, respectively. pR1m1 is a cDNA clone of RCNMV RNA1 (RNA1-m1) that does not generate SR1f in infected cells. First, 1 μg of SmaI-linearized pRC169c, pRC2|G, and pR1m1 were used as the template for *in vitro* transcription using MEGAscript T7 transcription kit (Invitrogen, AM1334), followed by DNase treatment according to the manufacturer’s protocol. The transcription reaction was carried out at 37°C for 4 h and DNase treatment was performed at 37°C for 30 min. Subsequently, RNA was purified using Zymo RNA Clean & Concentrator 5 kit (Zymo Research, R1015) and eluted in nuclease-free water.

### Virus inoculation in *Arabidopsis*.

*Arabidopsis* double knockout mutant line, *dcl2-1/dcl4-2t* (Germplasm, CS66078) (50), and the single-knockout mutant line, *xrn4-5* (Germplasm, CS68822) (52), were obtained from the *Arabidopsis* Biological Resource center (abrc.osu.edu), and the T-DNA insertion was verified by genotyping (Fig. 3 and 5A; see also Table S4 in File 1 in the supplemental material) (77). *Arabidopsis* Col-0 wild-type and the mutant lines were grown in growth chambers with 16 h of light at 24°C and 8 h of dark at 20°C. *Arabidopsis* plants were mechanically inoculated with RCNMV using the sap from RCNMV-infected N. benthamiana. First, 3-week-old N. benthamiana plants were mechanically inoculated with RCNMV RNA1 plus RNA2 (wt RCNMV inoculated) or RCNMV RNA1-m1 plus RNA2 (RCNMVΔSR1f inoculated) in 10 mM sodium phosphate buffer (pH 6.8). At 7 dpi, infected N. benthamiana leaves were ground in 10 mM sodium phosphate buffer (pH 6.8) with mortar and pestle, and the resulting sap was rubbed on two to three leaves per *Arabidopsis* plant using Q-tips and carborundum. Subsequently, new noninoculated *Arabidopsis* leaves were collected and pulverized. Total RNA was extracted using the TRIzol method (Invitrogen), and 2 to 5 μg of total RNA was used for Northern blot hybridization as previously described (49).

### Virus inoculation in *N. benthamiana* for RNA sequencing.

N. benthamiana plants were grown in a growth chamber with 16 h of light at 24°C and 8 h of dark at 20°C. At the four-leaf stage (two true leaves and two false leaves), the first and second true leaves were mechanically inoculated with (i) 10 mM sodium phosphate (pH 6.8) buffer (mock inoculated), (ii) 1 μg of RNA1 plus 1 μg of RNA2 in 10 mM sodium phosphate (pH 6.8) buffer per leaf (wt RCNMV inoculated), or (iii) 1 μg of RNA1-m1 plus 1 μg of RNA2 in 10 mM sodium phosphate (pH 6.8) buffer per leaf (RCNMVΔSR1f inoculated). Five plants were inoculated for each condition. Growth conditions were changed to 16 h of light at 20°C and 8 h of dark at 20°C. At 15 dpi, the seventh leaf was collected for each plant and pulverized in liquid nitrogen, followed by the addition of 1 ml of TRIzol (Invitrogen). Total RNA was extracted using Zymo Direct-zol miniprep columns (Zymo Research, R2051) and quantified using Qubit RNA HS assay kit (Invitrogen, Q32852). Subsequently, 5 μg of total RNA was treated with 1 μL of Turbo DNase (Invitrogen, AM2238) in a 50-μL reaction with 1× of Turbo DNase buffer at 37°C for 30 min, followed by the addition of 1 μL more of Turbo DNase, followed by incubation at 37°C for an additional 30 min, purification by Zymo RNA Clean & Concentrator 5 columns (Zymo Research, R1016), and quantification using a Qubit RNA HS assay kit (Invitrogen, Q32852). Total RNA integrity was verified using 1% agarose gel electrophoresis.

### cDNA synthesis and RT-PCR.

A RevertAid first-strand cDNA synthesis kit (Thermo Scientific, K1622) was used according to the manufacturer’s protocol with gene-specific primers. We mixed 1 μg of total RNA and 15 pmol of RCNMV-specific reverse primer (5′-GGGGTACCTAGCCGTTATAC-3′) in nuclease-free water to 12 μL, followed by incubation at 65°C for 5 min and transfer to ice, followed by the addition of 4 μL of reaction buffer, 1 μL of RiboLock, 2 μL of 10 mM deoxynucleoside triphosphate (dNTP), and 1 μL of RT enzyme. The reaction mixture was incubated at 42°C for 60 min, followed by enzyme deactivation at 70°C for 5 min. A 20-μL PCR mix was prepared with 10 μL of GoTaq G2 green master mix (Promega, M7823), 2 μL of cDNA template, and 200 nM concentrations (each) of forward (5′-AAGCGGGCCAGTAGAGTC-3′) and reverse (5′-CACAACATCCGCCAAAGAGG-3′) primers. The PCR conditions were as follows: 98°C (2 min); 25 cycles of 98°C (30 s), 65°C (20 s), 72°C (30 s); 72°C (2 min); and 4°C hold.

### RNA sequencing.

Next, 1 μg of DNase-treated total RNA from three biological replicates of mock-inoculated N. benthamiana and four biological replicates each of wt-RCNMV- and RCNMVΔSR1f-inoculated N. benthamiana were used for library preparation using a Zymo-seq RiboFree total RNA library prep kit (Zymo Research, R3000S). During the library preparation, the rRNA depletion was carried out for 45 min, and 10 cycles of library index PCR was performed using Zymo-Seq UDI primer set (Indexes 1 to 11; Zymo, D3008). Final libraries were quantified using a Qubit dsDNA HS assay kit (Invitrogen, Q32854), and the library quality was assessed using an Agilent bioanalyzer high-sensitivity DNA assay kit. Final libraries were sequenced using Zymo Research’s services on one high-output lane of Illumina HiSeq 1500 instrument with pair-ended 100-bp read length. The 11 RNA-seq samples were trimmed for adapters and quality using Trim galore 0.4.5 (https://github.com/FelixKrueger/TrimGalore). The N. benthamiana 1.0.1 genome and annotation was obtained from the Sol Genomics Network (78). Reads were mapped to the N. benthamiana (79) genome using Hisat2 2.1 (80) and processed using SAMtools 1.9 (81), and counts were obtained using featureCounts in the Subread 1.6 (82). Differential expression was computed using DESeq2 (53). A list of all DEGs can be found in the File 2 in the supplemental material. Principal-component analysis was performed using the regularized log-transformed data from DESeq2. For GO term enrichment analysis, the four DEG data set (wt RCNMV upregulated, wt RCNMV downregulated, RCNMVΔSR1f upregulated, and RCNMVΔSR1f downregulated) were used, and for KEGG pathway enrichment analysis, the two DEG data sets (i.e., the wt RCNMV versus mock and the RCNMVΔSR1f versus mock data sets) were used as input (http://kobas.cbi.pku.edu.cn/kobas3/genelist/) with the following parameters: type “Fasta protein sequence,” species “Arabidopsis thaliana,” pathway “KEGG pathway” or GO “GO,” statistical method “hypergeometric test/Fisher exact test,” and FDR correction method “Benjamini and Hochberg (1995).” The enriched GO terms with their adjusted *P* values were further used with Revigo (83) for visualization with the following parameters: allowed similarity “medium,” database “Arabidopsis thaliana,” and semantic similarity measure “SimRel.” The pathway involvement was visualized using KEGG Mapper (84). The functional annotation of the selected genes was determined using the “Niben101_annotation.proteins.wdesc.fasta” file from the Sol Genomics Network (78).

To analyze the RCNMV RNA abundance, the adapter trimmed reads were mapped to RCNMV RNA1/RNA1-m1 and RNA2 using bowtie2 (85) with the “-sensitive-local” option. To obtain alignment information individually for positive and negative strands of RCNMV RNAs, the alignment file was split according to their flag information using SAMtools. Subsequently, the Salmon tool (86) was used to quantify the number of reads mapping to positive and negative strands of the RCNMV genome. The read counts were normalized by dividing the read count to the DESeq2 scaling factors to account for the sequencing depth. The scaling factors were obtained using DESeq2 with the count table of reads mapping to the N. benthamiana genome and RCNMV genome as input. To obtain read coverage on the RCNMV genome, the SAM files were converted to BAM file format, sorted, and indexed using SAMtools. Subsequently, the bamCoverage function in deepTools 2.5.2 (87) was used with “-scaleFactor” option with the reciprocal of DESeq2 scaling factors for each sample. In addition, the “-filterRNAstrand” option is set as “forward” or “reverse” to get read coverage on the positive and negative strands of RCNMV separately. Geometric mean of the scaled number of reads that mapped to each nucleotide position of RCNMV genome in the output bedgraph file was plotted in the Fig. 12.

### *In vitro* translation.

Wheat germ extract (WGE; Promega, L4380) was used for *in vitro* translation. Triplicates of 389 ng of each (25 nM final RNA concentration) of *in vitro*-transcribed RCNMV RNA1 or RNA1-m1 in 3.75 μL of water were incubated at 67°C for 10 min and transferred to ice, followed by the addition of 1 μL of amino acid mix (without methionine), 1 μL of 1 M potassium acetate, 0.5 μL of EasyTag l-[^35^S]methionine (Perkin-Elmer, NEG709A), and 6.25 μL of WGE. The reaction mixture was incubated at 25°C for 30 min, and translation was terminated by transferring the tubes to ice. To the 12.5-μL reaction, 3.2 μL of NuPAGE 4× LDS sample buffer (Invitrogen, NP0007) and 1.5 μL of NuPAGE 10× sample reducing agent (Invitrogen NP0009) were added, followed by incubation at 70°C for 10 min, and a 15-μL reaction was run in NuPAGE Novex 4 to 12% Bis-Tris gel (Invitrogen, NP0322BOX) with 1× NuPAGE MES SDS running buffer (Invitrogen, NP002) at 200 V for 40 min. The gel was washed three times with water for 5 min, once with fixing solution (50% methanol plus 7% acetic acid) for 15 min, and three times with water for 5 min. All washing steps were carried out at room temperature. The dried gel was imaged by autoradiography using a Bio-Rad PharosFX Plus molecular imager.

### qRT-PCR.

First, 1 μg of DNase-treated total RNA (the same RNA used for RNA-seq) from two biological replicates of mock-inoculated N. benthamiana samples and four biological replicates each of wt-RCNMV- and RCNMVΔSR1f-inoculated N. benthamiana samples were reverse transcribed using a RevertAid first-strand cDNA synthesis kit (Thermo Scientific, K1622) according to the manufacturer’s protocol with random hexamers. The resulting cDNA was diluted 10- and 20-fold to quantify the abundance of transcripts from N. benthamiana and RCNMV, respectively. A 10-μL qPCR was prepared with 1× iQ SYBR green Supermix (Bio-Rad 1708880), 300 nM (each) forward and reverse primer, and 1 μL of diluted cDNA template. The qPCR runs were carried out in 384-well plates with three technical replicates per sample in a Bio-Rad CFX384 system with the following reaction conditions: 95°C for 3 min (polymerase activation and DNA denaturation), 40 cycles of 95°C for 10 s (denaturation), and 60°C for 60 s (annealing, extension/plate reading), followed by melting-curve analysis (55 to 95°C, 0.5°C increments, 5 s). NbPP2A and NbL23 genes were used as reference genes to normalize the abundance of N. benthamiana and RCNMV RNAs (88). Prior to using these as reference genes, we verified their consistent expression between our experimental conditions. The primer efficiency calculation, ΔΔ*C_T_* calculation, and statistical analysis were performed using the Bio Rad CFX manager software. The reference gene primer sequences were obtained from Liu et al. (88), the NbPR1 primer sequences were obtained from Obrępalska-Stęplowska et al. (89), and all of the remaining primers were designed using the primer3 tool (https://primer3.ut.ee/) (90, 91). To verify the specificity of the primers and determine the Unigene IDs that would be amplified by the primers, we used the Primer-BLAST tool (https://www.ncbi.nlm.nih.gov/tools/primer-blast/) against the N. benthamiana 1.0.1 transcript sequences obtained from the Sol Genomics Network (78) with default parameters. The Unigene IDs that gave the expected amplicon size had a maximum of two-base mismatches within the primers, but no mismatch at the last three bases in the 3′ end of the primers was considered or is included in Fig. 9. All the primers were synthesized by Integrated DNA Technologies and purified by standard desalting. qRT-PCR primer sequences are listed in Table S3 in File 1 in the supplemental material.

### Data availability.

The raw sequencing fastq files, RCNMV genome-mapped read counts and N. benthamiana genome-mapped fragment counts were deposited in the NCBI Gene Expression Omnibus database under accession number GSE178909.
