# Supplementary material for: Effects of the Noncoding Subgenomic RNA of Red Clover Necrotic Mosaic Virus in Virus Infection
Source: J Virol. 2022 Feb 9;96(3):e01815-21. doi: 10.1128/jvi.01815-21 (PMC8826918; doi:10.1128/jvi.01815-21)
Supplement: Supplemental file 1 — Table S1 to S4 and Fig. S1 to S5. Download jvi.01815-21-s0001.pdf, PDF file, 2.9 MB [file jvi.01815-21-s0001.pdf]

Supplementary File 1

Supplementary tables and figures

Supplementary table S1. List of differentially expressed LRR-RLKs/RLPs genes.

	Wt RCNMV vs. mock		RCNMVΔSR1f vs. mock		
**Unigene ID**	**Log_2FC**	**adj. p**	**Log_2FC**	**adj.p**	**Type**
Niben101Scf03816g01001	2.72	2.09E-15	0.16	0.916	
Niben101Scf04099g05004	2.82	2.47E-12	0.11	0.955	
Niben101Scf05961g02036	1.54	2.05E-10	-0.07	0.943	
Niben101Scf06509g02006	3.94	3.12E-10	0.92	NA	
Niben101Scf05928g03007	5.45	3.10E-09	1.33	NA	
Niben101Scf20124g00014	2.33	5.33E-08	-0.06	0.970	
Niben101Scf00245g00006	7.43	3.83E-07	1.12	NA	
Niben101Scf00160g06027	5.85	3.92E-06	1.60	NA	
Niben101Scf04709g00016	1.41	5.42E-06	0.17	0.803	
Niben101Scf00541g05003	-1.65	5.62E-06	-0.71	0.231	
Niben101Scf01148g00007	1.32	1.15E-05	-0.09	0.923	
Niben101Scf04053g03008	2.04	1.51E-05	-0.39	0.789	
Niben101Scf03685g00003	1.87	1.89E-05	-0.28	0.793	
Niben101Scf03021g00010	1.38	2.55E-05	-0.04	0.971	
Niben101Scf02417g01010	2.05	6.48E-05	0.37	NA	
Niben101Scf09296g00007	-1.91	1.45E-04	-0.17	0.886	
Niben101Scf00985g06002	-1.55	1.72E-04	0.01	0.993	
Niben101Scf01237g07019	-2.06	2.62E-04	0.34	0.596	
Niben101Scf00953g00004	1.69	2.73E-04	0.51	0.692	LRR-RLK
Niben101Scf07323g01002	-1.15	3.02E-04	-0.22	0.591	
Niben101Scf03251g00016	-1.25	7.48E-04	-1.00	0.487	
Niben101Scf12414g01006	-1.28	0.001	-0.28	0.624	
Niben101Scf08564g00001	1.65	0.002	0.03	0.988	
Niben101Scf01225g04031	1.49	0.006	-0.21	0.903	
Niben101Scf05437g06022	5.02	0.007	2.95	NA	
Niben101Scf04377g07005	1.03	0.008	0.63	0.293	
Niben101Scf14708g00025	1.36	0.008	0.25	0.860	
Niben101Scf01278g09008	1.23	0.013	-0.27	0.734	
Niben101Scf09811g00006	1.18	0.014	0.06	0.967	
Niben101Scf03098g00011	-1.39	0.019	0.23	0.854	
Niben101Scf10381g03006	4.63	0.020	2.34	NA	
Niben101Scf01519g01007	1.00	0.025	-0.23	0.721	
Niben101Scf09774g00001	-1.10	0.025	-0.73	0.278	
Niben101Scf17094g01001	-1.05	0.031	0.10	0.928	
Niben101Scf04609g00013	4.09	0.033	1.17	NA	
Niben101Scf04430g01006	-1.05	0.035	-0.31	0.743	
Niben101Scf02646g03004	1.26	0.04997	0.36	0.750	
Niben101Scf00745g02015	1.70	2.07E-04	0.27	0.813	
Niben101Scf07123g01015	7.03	5.51E-34	2.42	0.140	
Niben101Scf02072g01013	3.93	3.30E-22	0.88	0.428	
Niben101Scf02252g01032	4.06	1.67E-20	0.44	0.794	
Niben101Scf03240g00007	2.33	2.05E-08	0.45	0.709	LRR-RLP
Niben101Scf03202g08006	2.43	8.18E-08	1.01	0.780	
Niben101Scf03925g01010	3.31	1.34E-07	0.20	NA	

Niben101Scf13842g01001	2.64	5.95E-06	0.93	0.509
Niben101Scf11676g00001	3.06	2.04E-04	-0.03	NA
Niben101Scf00714g06002	5.92	2.61E-04	NA	NA
Niben101Scf05982g00007	2.21	0.002	-0.80	NA
Niben101Scf03202g07009	5.31	0.003	2.30	NA
Niben101Scf00975g01015	4.36	0.005	0.75	NA
Niben101Scf07123g01020	1.98	0.006	1.03	NA
Niben101Scf02646g02009	1.42	0.007	-0.11	0.946
Niben101Scf02854g10010	1.30	0.0473	-0.85	NA

FC: Fold change, adj. p: corrected p-values, NA: not available in DESeq2 output

Supplementary File 1

Supplementary table S2. Selected differentially expressed genes that are known to be co-opted by Tombusvirids.

			Wt RCNMV vs. mock		RCNMVΔSR1f vs. mock	
**Gene**	**Protein**	**Unigene ID**	**Log_2FC**	**adj. p**	**Log_2FC**	**adj. p**
Rboh	Respiratory burst oxidase homolog protein A	Niben101Scf10840g01010	1.35	1.52E-03	0.58	0.27
CDPK	Calcium-dependent protein kinase	Niben101Scf15752g00002	1.95	1.92E-06	0.63	0.37
		Niben101Scf00800g02008	2.05	6.17E-10	0.63	0.42
		Niben101Scf09296g01009	1.00	8.89E-03	0.47	0.45
		Niben101Scf09345g00003	1.43	7.89E-04	0.80	0.52
		Niben101Scf03377g04002	1.03	3.27E-04	0.38	0.59
		Niben101Scf05534g01007	2.15	5.29E-18	0.19	0.87
		Niben101Scf00539g05012	2.38	6.09E-08	0.18	0.92
		Niben101Scf01166g14001	2.41	7.41E-03	0.14	0.94
		Niben101Scf00083g00009	1.01	5.26E-04	-0.01	0.99
		Niben101Scf17776g00008	2.65	0.0213	0.03	NA
		Niben101Scf04216g07017	2.12	0.0317	0.05	NA
PLD	Phospholipase D	Niben101Scf02465g00004	1.81	2.46E-07	-0.07	0.95
		Niben101Scf00539g07022	2.24	7.01E-05	0.85	NA
		Niben101Scf03930g01018	6.05	1.20E-04	3.96	NA
HSP70	Heat shock protein 70	Niben101Scf04364g01014	1.04	1.75E-04	0.24	0.63
		Niben101Scf02771g01007	1.05	1.93E-04	0.29	0.41
		Niben101Scf07275g02012	1.36	3.18E-03	0.28	0.72
		Niben101Scf00449g06008	1.55	1.39E-04	-0.52	0.31
		Niben101Scf04490g00001	2.05	0.0473	0.41	NA
		Niben101Scf13703g01006	3.74	7.12E-04	2.48	NA
HSP90	Heat shock protein 90	Niben101Scf04331g09018	1.03	1.43E-03	0.55	0.48
		Niben101Scf01027g00003	1.38	0.0102	0.86	0.30
		Niben101Scf06890g01022	1.52	6.80E-11	0.74	0.30
		Niben101Scf27914g00006	1.60	2.92E-12	0.50	0.39
Arf	ADP-ribosylation factor	Niben101Scf01063g07014	1.24	6.30E-04	-0.19	0.89
ORP	Oxysterol-binding protein-related protein	Niben101Scf00126g06015	1.00	3.86E-03	0.56	0.22
		Niben101Scf02429g00001	1.03	1.95E-03	0.42	0.47
VAP	Vesicle-associated membrane protein-associated protein	Niben101Scf04122g05011	1.18	1.50E-04	-0.38	0.71
BRO1	Vacuolar protein-sorting protein bro1	Niben101Scf12308g00014	1.234	1.22E-06	0.57	0.19
		Niben101Scf04109g03009	1.326	1.22E-06	0.56	0.25
		Niben101Scf04973g02006	1.78	4.53E-05	0.46	0.44

FC: Fold change, adj. p: corrected p-values, NA: not available in DESeq2 output

Supplementary File 1

Supplementary table S3. List of primers used for qRT-PCR.

Primer Name	Sequence (5'-3') *	Amplicon Size (bp)	Efficiency#	Miscellaneous
RCNMV_1457_FP<br>RCNMV_1574_RP	CAACAGGGCTCAAGGGAGAG<br>GAATTTGAGGGCATCGACGC	118	104.14 %	Amplifies RCNMV RNA 1
RC_R2_479_FP<br>RC_R2_668_RP	AGGGTGCGAATCACGAATAC<br>ACTGCACGTAGGCTTCCACT	190	100.21 %	Amplifies RCNMV RNA 2
Nbenth_PP2A_FP<br>Nbenth_PP2A_RP	GACCCTGATGTTGATGTTCGCT<br>GAGGGATTTGAAGAGAGATTTC	123	92.76-<br>106.91 %	Protein phosphatase 2A;<br>TC21939 (At1g13320) R
Nbenth_L23_FP<br>Nbenth_L23_RP	AAGGATGCCGTGAAGAAGATGT<br>GCATCGTAGTCAGGAGTCAACC	110	83.09 -<br>109.12 %	60S ribosomal protein;<br>TC19271 (At2g39460) R
NbPR1_FP<br>NbPR1_RP	GGATGCCCATAACACAGCTC<br>GCTAGGTTTTCGCCGTATTG	150	93.52 %	Amplifies PR1 genes
NbPR2_FP<br>NbPR2_RP	GATGCCCTTTTGGATTCTATG<br>TTGCTGCAGAGTTTCCTTCA	109	86.91 %	Amplifies PR2 genes
NbABF_FP<br>NbABF_RP	TTGGGAAGTCCTGGAATGAG<br>TAACAGCTCCGGCTCCTAAA	130	102.86 %	Amplifies ABF genes
NbWrky_FP<br>NbWrky_RP	TCTTTAGCCGTCCAGCCTTA<br>CGTCGTCGAAATCATCTCCT	169	93.86 %	Amplifies NbWRKY25/33 genes
NbRboh_FP<br>NbRboh_RP	TCAAGAACTCAAGCGGGTCT<br>GACCAACAAGCAGCAAGACA	176	107.75 %	Amplifies NbRboh genes
NbJAZ_FP<br>NbJAZ_RP	ACATGAGCAATCCCTCCAAC<br>GACCGTCCCACCATAGAAGA	218	104.06 %	Amplifies NbJAZ genes
NbPro-inh_FP<br>NbPro-inh_RP	GCTTTCTTGCTCCTTGCATC<br>GTTCAGGCCATGATTGCTTT	175	108.66 %	Amplifies NbPI genes

Primer efficiency was calculated using standard curve with 5 points of dilution with each run.

R Used as reference genes.

* The primer sequence for NbPP2A, and NbL23 was by obtained from Liu et al. (2012) (1). The primer sequence for NbPR1 gene was obtained from Obrępalska-Stęplowska et al. (2018) (2). All the remaining primers were designed using the primer3 tool (https://primer3.ut.ee/).

Supplementary File 1

Supplementary table S4. List of primers used for genotyping Arabidopsis mutants.

<table>
<tr><th>Primer Name</th><th>Sequence (5’-3’) *</th><th colspan="2">Amplicon Size (bp)</th></tr>
<tr><td>at_xrn4-5_for</td><td>GTTTCTTGGTTGTTGCAGCTC</td><td rowspan="2">1164</td><td></td></tr>
<tr><td>at_xrn4-5_rev</td><td>TCATGACGAATTCCTTTGAGG</td><td rowspan="2">593-893</td></tr>
<tr><td>pDAP101.LB3#</td><td>TAGCATCTGAATTTCATAACCAATCTCGATACAC</td><td></td></tr>
<tr><td>at_dcl2-1_for</td><td>TGAATCATCTGGAAGAGGTGG</td><td rowspan="2">1060</td><td></td></tr>
<tr><td>at_dcl2-1_rev</td><td>CTTCACAGGAGTTTTTGGCTG</td><td rowspan="2">459-759</td></tr>
<tr><td>pROK2_LBb1.3#</td><td>ATTTTGCCGATTTCGGAAC</td><td rowspan="2">546-846</td></tr>
<tr><td>at_dcl3-1_rev</td><td>TGGAAAAGTTTGCTACAACGG</td><td rowspan="2">1085</td></tr>
<tr><td>at_dcl3-1_for</td><td>ACAGGTAACCTTGCCATGTTG</td><td></td></tr>
<tr><td>at_dcl4-2t_for</td><td>AAGAGAACTTTTGCCGAAAGC</td><td rowspan="2">1222</td><td></td></tr>
<tr><td>at_dcl4-2t_rev</td><td>TTTGCCAGTCTTACAAGTGGG</td><td rowspan="2">572-872</td></tr>
<tr><td>pAC161_8474#</td><td>ATAATAACGCTGCGGACATCTACATTTT</td><td></td></tr>
<tr><td colspan="4"># Primers for T-DNA vector sequences
* Primers were designed using T-DNA express (http://signal.salk.edu/tabout.html) according to Leicht and Cheng (2009) and O’Malley et al. (2015) (3-4).</td></tr>
</table>

Supplementary File 1

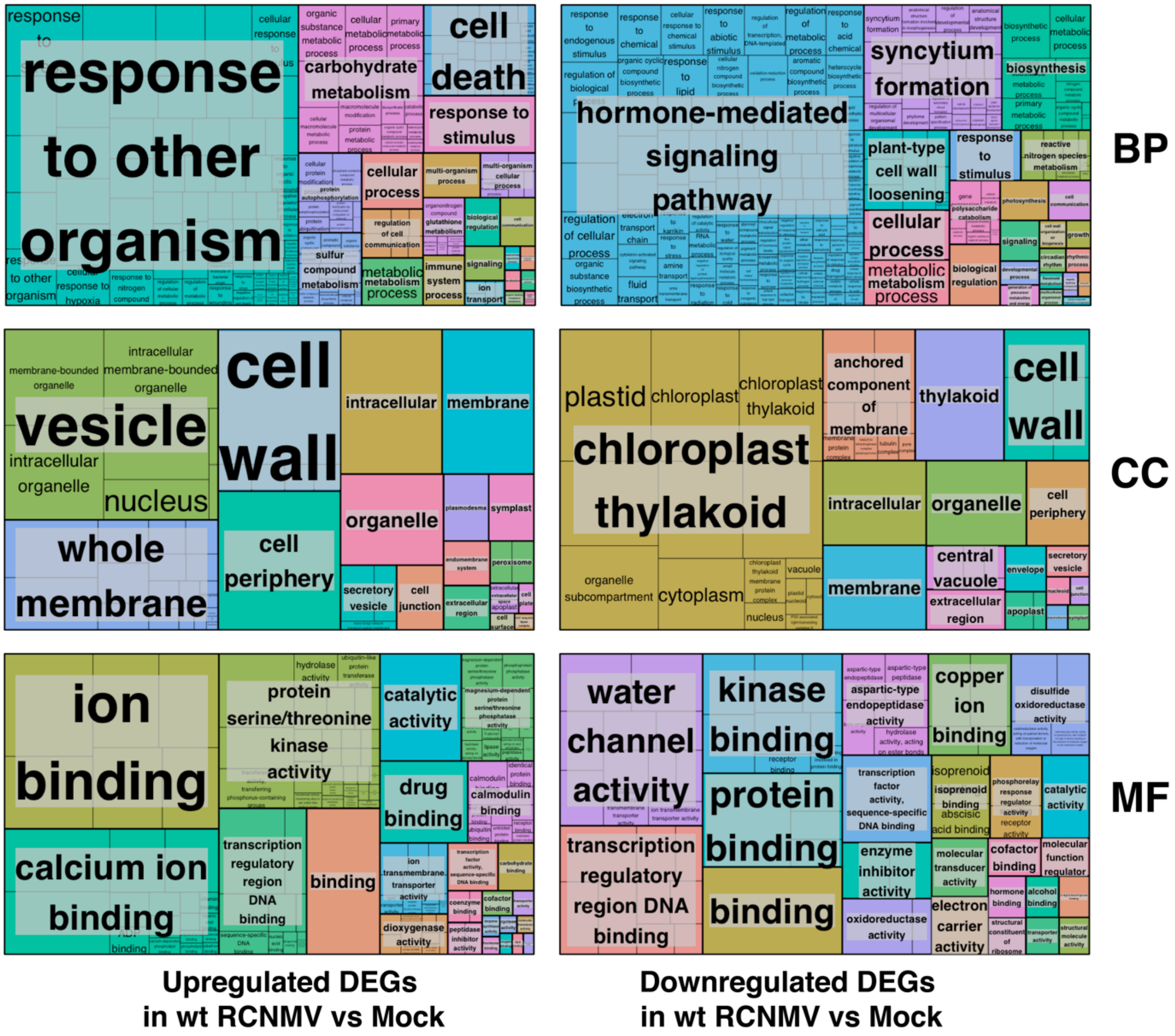

Supplementary Figure S1. TreeMap view of enriched GO terms in wt RCNMV vs mock. The upregulated and downregulated genes were separately used to find the enriched GO terms. The absolute $\log_{10}$ p-value determines the size of the rectangles. BP: Biological process, CC: Cellular compartment, MF: Molecular function.

Supplementary File 1

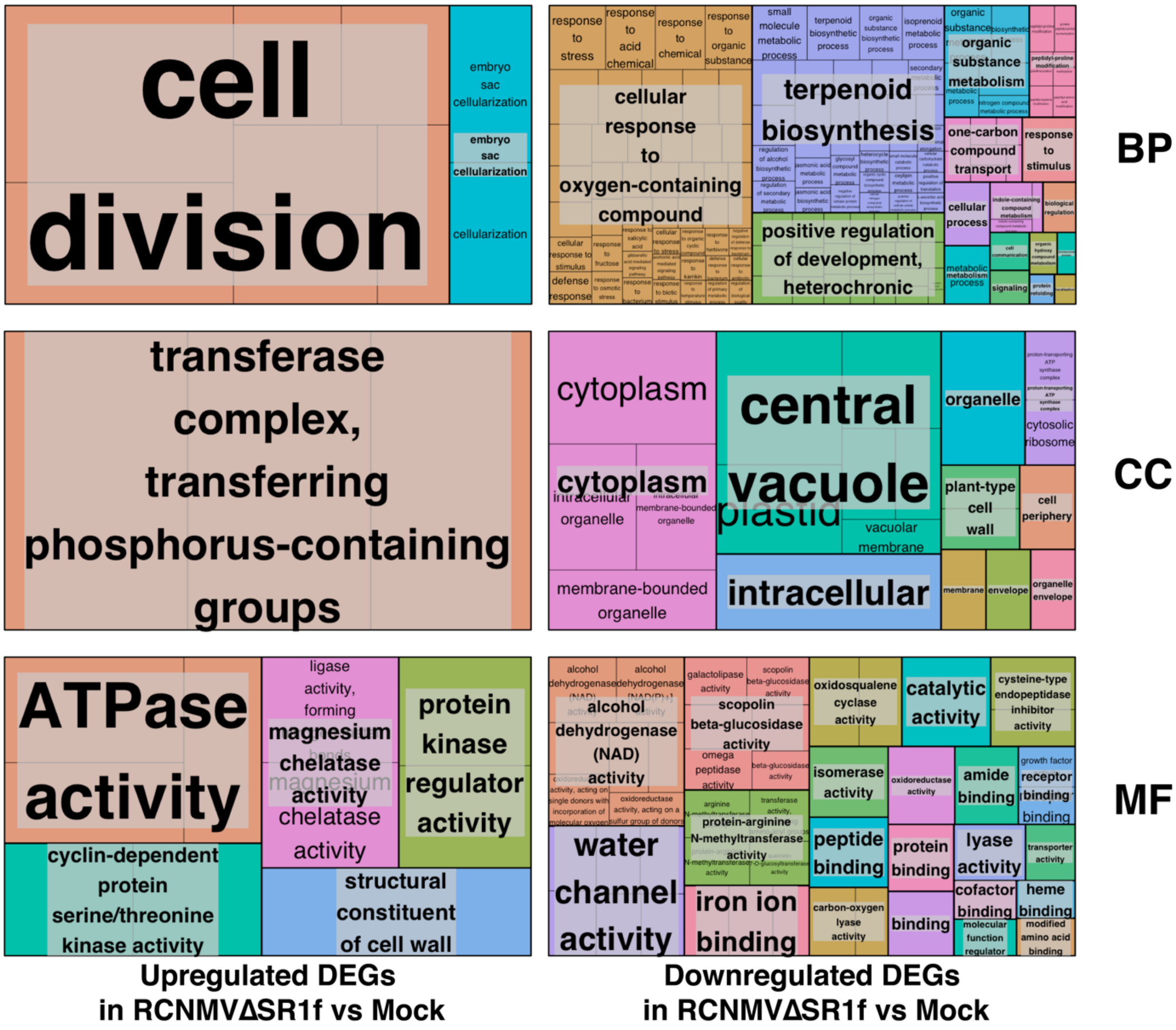

Supplementary Figure S2. TreeMap view of enriched GO terms in RCNMVΔSR1f vs mock. The upregulated and downregulated genes were separately used to find the enriched GO terms. The absolute $\log_{10}$ p-value determines the size of the rectangles. BP: Biological process, CC: Cellular compartment, MF: Molecular function.

Supplementary File 1

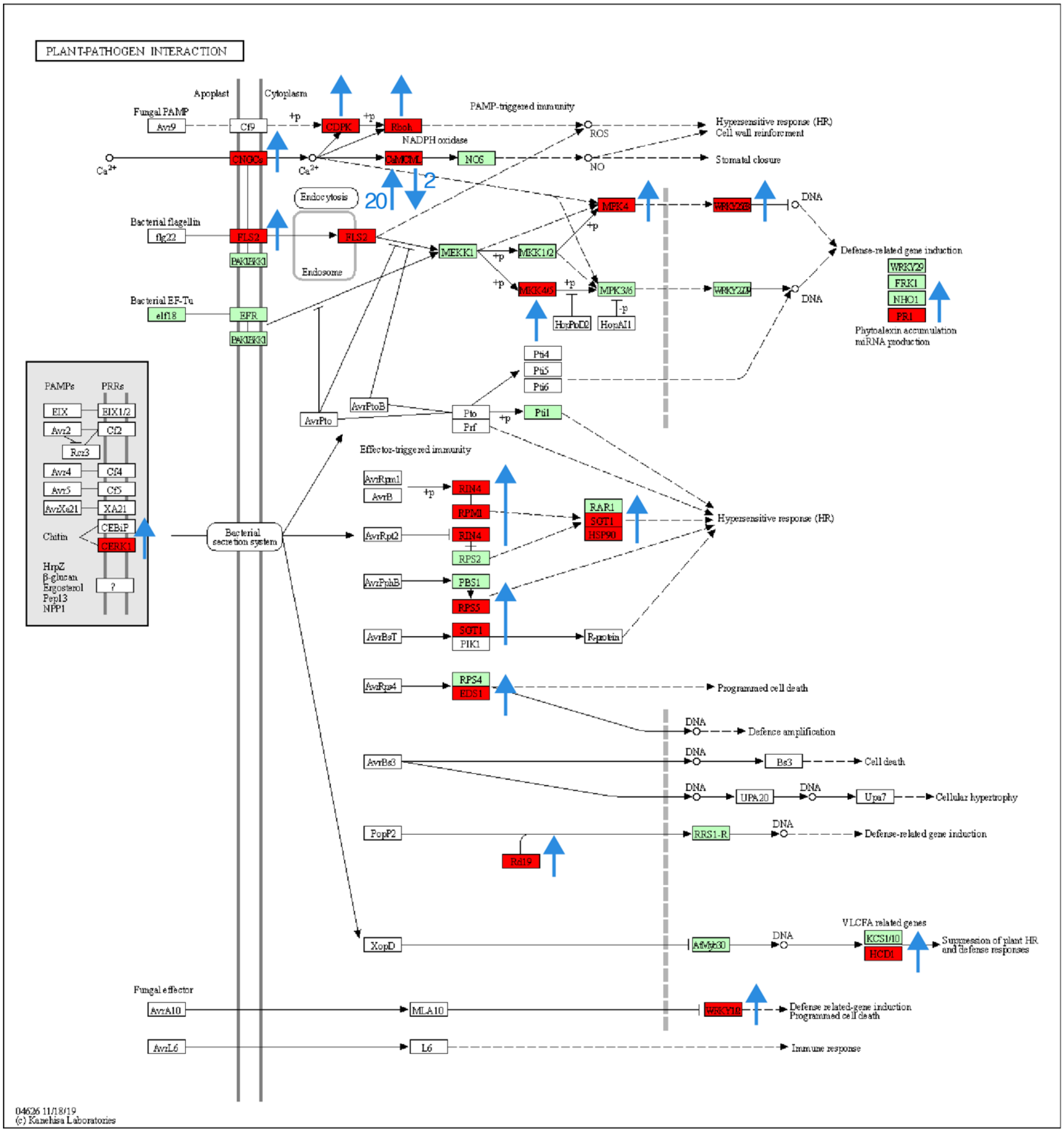

Supplementary Figure S3. DEGs in wt RCNMV vs mock data associated with Plant-Pathogen interaction pathway. The red boxes refers to genes that were differentially expressed. The direction of blue arrows depict if the genes were up- or down-regulated. The numbers beside the arrows refer to the number of unigenes that were regulated in the direction of the arrow. Arrows without the numbers means all the unigenes were regulated in the same direction.

Supplementary File 1

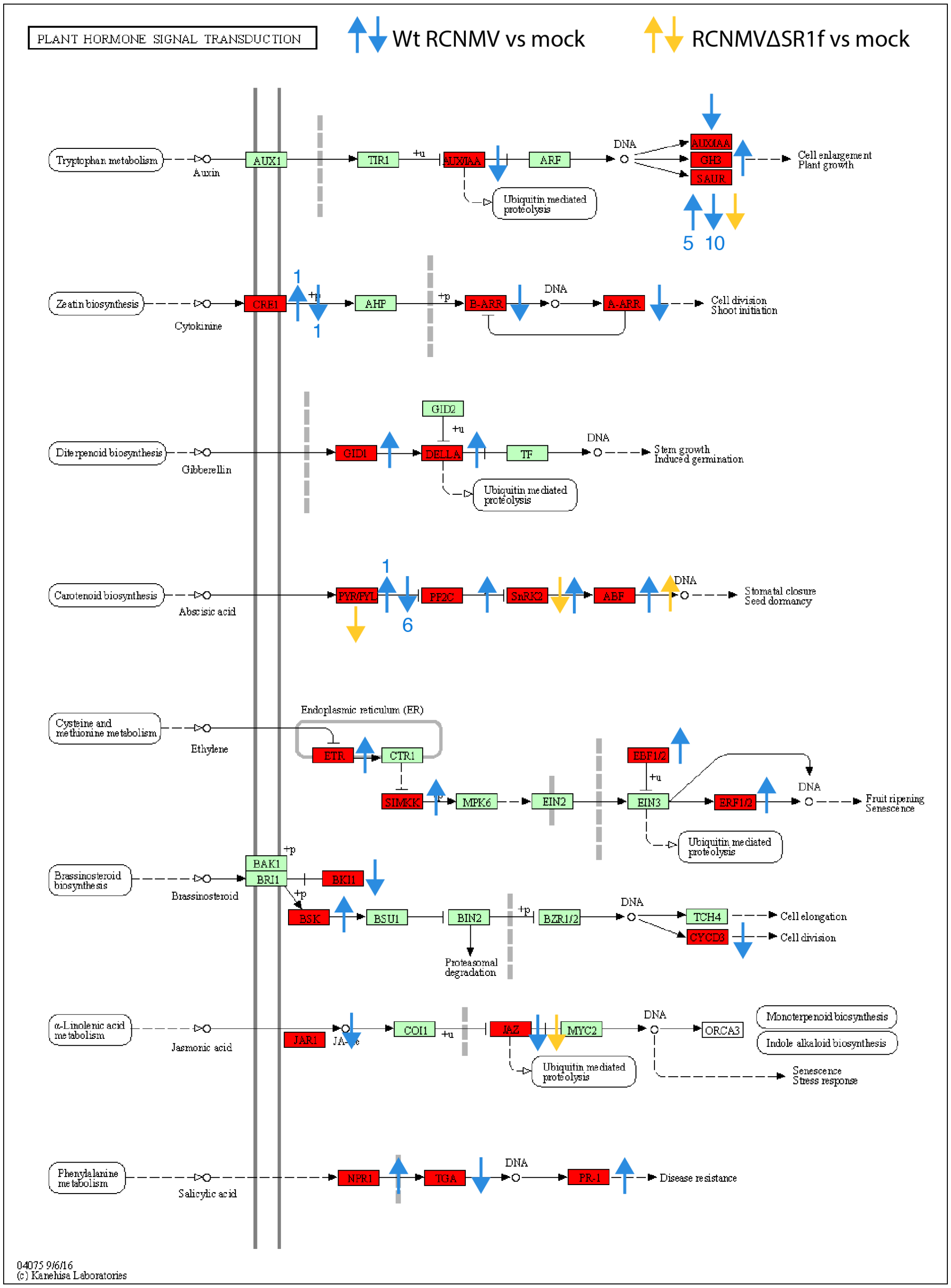

Supplementary Figure S4. DEGs in wt RCNMV vs mock data and RCNMVΔSR1f vs mock data associated with Plant hormone signal transduction pathway. The red boxes refers to genes that were differentially expressed. The direction of blue and yellow arrows depict the direction of regulation in wt RCNMV vs mock data and RCNMVΔSR1f vs mock data, respectively. The numbers beside the arrows refer to the number of unigenes that were regulated in the direction of the arrow. Arrows without the numbers means all the unigenes were regulated in the same direction.

Supplementary File 1

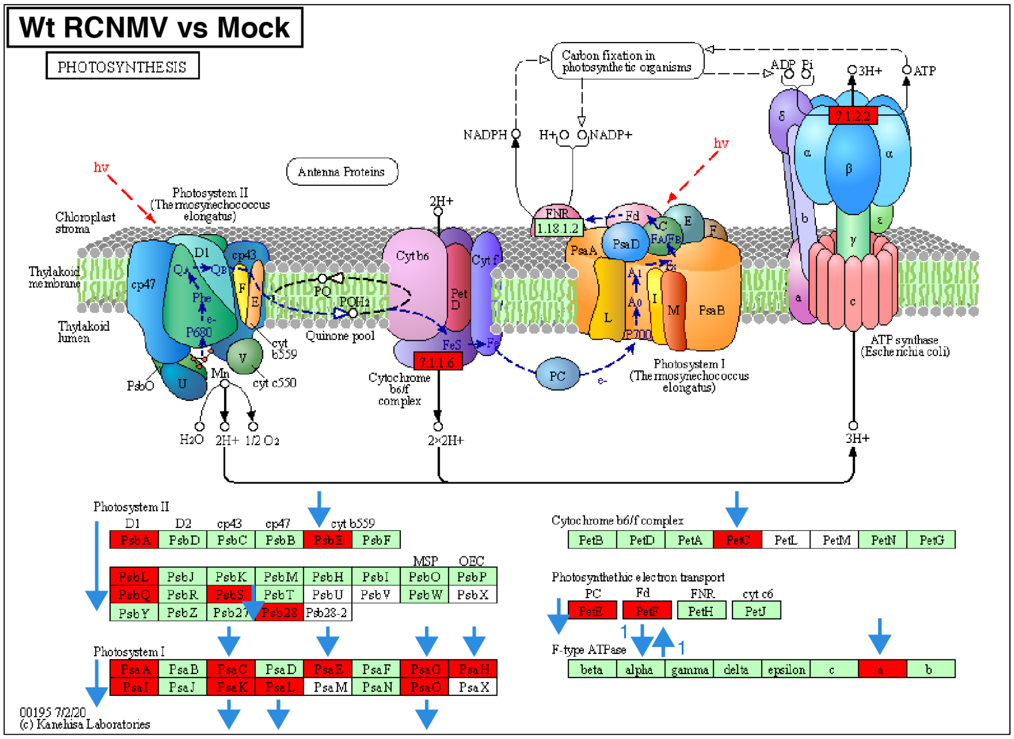

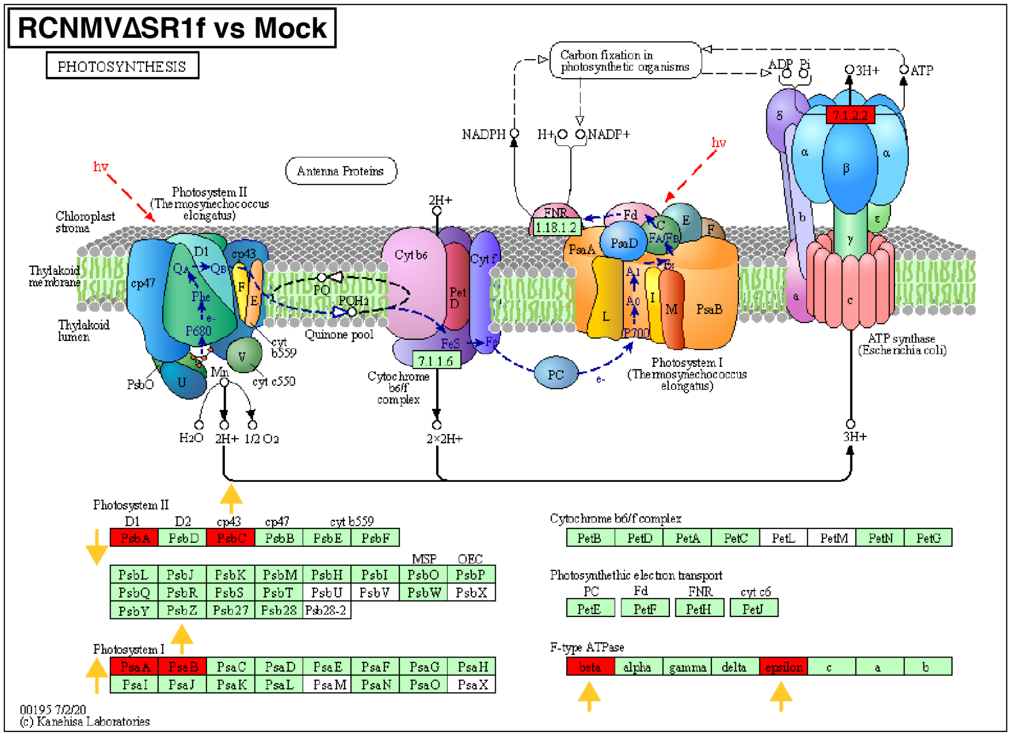

Supplementary Figure S5. DEGs in wt RCNMV vs mock data and RCNMVΔSR1f vs mock data associated with Photosynthesis pathway. The red boxes refers to genes that were differentially expressed. The direction of blue and yellow arrows depict the direction of regulation in wt RCNMV vs mock data and RCNMVΔSR1f vs mock data, respectively. The numbers beside the arrows refer to the number of unigenes that were regulated in the direction of the arrow. Arrows without the numbers means all the unigenes were regulated in the same direction.

Supplementary File 1

References:

1) Liu D, Shi L, Han C, Yu J, Li D, Zhang Y. 2012. Validation of Reference Genes for Gene Expression Studies in Virus-Infected Nicotiana benthamiana Using Quantitative Real-Time PCR ed. B.A. Vinatzer. PLoS One 7: e46451.

2) Obrępalska-Stęplowska A, Zmienko A, Wrzesińska B, Goralski M, Figlerowicz M, Zyprych-Walczak J, Siatkowski I, Pospieszny H. 2018. The Defense Response of Nicotiana benthamiana to Peanut Stunt Virus Infection in the Presence of Symptom Exacerbating Satellite RNA. Viruses 10: 449.

3) Leicht BG and CL Cheng. 2009. Reverse genetics: Identification of Arabidopsis lines with T-DNA insertions in a gene of interest. Pages 282-290, in Tested Studies for Laboratory Teaching, Volume 30 (K.L. Clase, Editor). Proceedings of the 30th Workshop/Conference of the Association for Biology Laboratory Education (ABLE), 403 pages.
(https://studylib.net/doc/18550265/bringing-the-lab-into-your-lectures)
(https://www.ableweb.org/biologylabs/wp-content/uploads/volumes/vol-30/018.pdf)

4) O'Malley RC, Barragan CC, Ecker JR. 2015. A User's Guide to the Arabidopsis T-DNA Insertion Mutant Collections. In Methods in molecular biology (Clifton, N.J.), Vol. 1284 of, pp. 323–342, Howard Hughes Medical Institute.
